# Molecular Visualization of Early‐Stage Acute Kidney Injury with a DNA Framework Nanodevice

**DOI:** 10.1002/advs.202105947

**Published:** 2022-05-04

**Authors:** Fei Ding, Shuangye Zhang, Suyu Liu, Jing Feng, Jiang Li, Qian Li, Zhilei Ge, Xiaolei Zuo, Chunhai Fan, Qiang Xia

**Affiliations:** ^1^ Institute of Molecular Medicine Department of Liver Surgery Shanghai Key Laboratory for Nucleic Acid Chemistry and Nanomedicine Renji Hospital School of Medicine Shanghai Jiao Tong University Shanghai 200127 China; ^2^ School of Chemistry and Chemical Engineering Frontiers Science Center for Transformative Molecules and National Center for Translational Medicine Shanghai Jiao Tong University Shanghai 200240 China; ^3^ Southern Medical University Affiliated Fengxian Hospital The Second Affiliated Hospital of the Chinese University of Hong Kong (Shenzhen) Shenzhen 518172 China; ^4^ Bioimaging Center Shanghai Synchrotron Radiation Facility Zhangjiang Laboratory, Shanghai Advanced Research Institute Chinese Academy of Sciences Shanghai 201210 China; ^5^ WLA Laboratories Shanghai 201203 China

**Keywords:** acute kidney injury, DNA nanodevice, early diagnosis, kidney injury molecule‐1, tetrahedral DNA framework

## Abstract

DNA nanomachines with artificial intelligence have attracted great interest, which may open a new era of precision medicine. However, their in vivo behavior, including early diagnosis and therapeutic effect are limited by their targeting efficiency. Here, a tetrahedral DNA framework (TDF)‐based nanodevice for in vivo near‐infrared (NIR) diagnosis of early‐stage AKI is developed. This nanodevice comprises three functional modules: a size‐tunable TDF nanostructure as kidney‐targeting vehicle, a binding module for the biomarker kidney injury molecule‐1 (Kim‐1), and a NIR signaling module. The cooperation of these modules allows the nanodevice to be selectively accumulated in injured kidney tissues with high Kim‐1 level, generating strong NIR fluorescence; whereas the nanodevice with the proper size can be rapidly cleared in healthy kidneys to minimize the background. By using this nanodevice, the early diagnosis of AKI onset is demonstrated at least 6 h ahead of Kim‐1 urinalysis, or 12 h ahead of blood detection. It is envisioned that this TDF‐based nanodevice may have implications for the early diagnosis of AKI and other kidney diseases.

## Graphical abstract

A tetrahedral DNA framework‐based nanodevice integrating three functionalities allows real‐time tracking of the dynamic variation of kidney injury molecule‐1 in high contrast manner, and can be used as imaging agent and urinalysis tracers to non‐invasively diagnose the onset of acute kidney injury earlier than that can be detected by the conventional in vitro assays.

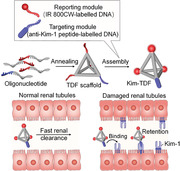

## 1 Introduction

Nanodevices are promising vehicles to deliver agents such as drugs, contrast agents, and gene editors to a specific diseased tissue or cell for diagnosis and therapy.^[^ ^1^ ^]^ In recent decades, DNA has been shown to be an intelligent building block to fabricate structurally precise nanodevices integrating versatile functionalities owing to its molecular programmability, thus DNA nanotechnology‐enabled nanodevices permit pathology‐specific molecular intelligence.^[^ ^2^ ^]^ However, the in vivo environment is complex and compartmentalized with barriers at different levels, which largely constrains the targeting ability of DNA nanodevices and thus weakens their performance in vivo.^[^ ^3^ ^]^

Recent studies have shown that compact DNA nanostructures exhibit inherent kidney‐targeting ability, making them promising therapeutic agents for renal diseases such as acute kidney injury (AKI).^[^ ^4^ ^]^ Moreover, the renal‐clearable tetrahedral DNA framework (TDF) enables a noninvasive evaluation of kidney function by measuring its renal clearance kinetics.^[^ ^5^ ^]^ Nevertheless, owing to the renal compensatory, this strategy cannot timely detect slight histopathologic damages in kidney.^[^ ^6^ ^]^ Transmembrane glycoprotein kidney injury molecule‐1 (Kim‐1) as a specifically upregulated protein in kidney at the early stage of AKI, can serve as a reliable biomarker for AKI diagnosis.^[^ ^7^ ^]^ Yet conventional urinalysis of Kim‐1 generally suffers a delay of hours due to the time‐consuming urine production process.^[^ ^7^, ^8^ ^]^ Therefore, the intelligence DNA nanodevice provides opportunities for early diagnosis of AKI by longitudinally tracking Kim‐1.

Here, we develop a tetrahedral DNA framework‐based nanodevice (Kim‐TDF) for the near‐infrared (NIR) imaging identification of AKI kidneys in vivo with enhanced AKI‐to‐normal contrast. This nanodevice is integrated from three functional modules with stoichiometric precision:^[^ ^9^ ^]^ (1) a kidney‐targeting tetrahedral DNA framework with size‐dependent renal clearance kinetics, serving as the scaffold of the nanodevice; (2) a Kim‐1‐targeting module for the binding of Kim‐1 molecules in injured kidneys; (3) NIR fluorophores with high photostability for imaging signaling. This nanodevice can be selectively accumulated in injured kidneys with high Kim‐1 level, generating strong NIR fluorescence; whereas, in healthy kidneys, the nanodevice can be rapidly cleared, minimizing the background and thus enabling a high AKI‐to‐normal ratio.

## 2 Results and Discussion

### 2.1 Design and Synthesis of Kim‐TDF Nanodevices

**Figure 1:**
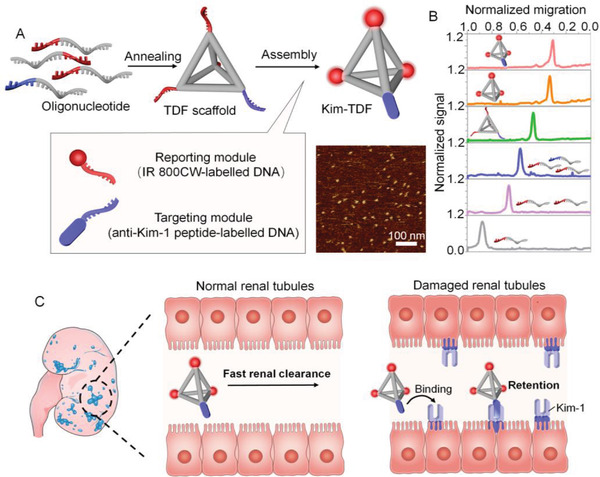
Design and characterization of the engineering TDF nanodevice (Kim‐TDF). A) Schematic illustration of the construction of the Kim‐TDF. The Kim‐TDF_17_ were analyzed by AFM. B) Characterization of Kim‐TDF_17_ by native PAGE gel electrophoresis. C) The schematic illustration of fluorescence imaging of Kim‐1 with enhanced AKI‐to‐normal contrast by using Kim‐TDF nanodevice.

We first describe the design principle of DNA framework‐based nanodevice comprising three functional modules (**Figure** **1**A). First is the kidney‐targeting DNA scaffold, a tetrahedral DNA framework (TDF) structure, carrying four overhangs as docking sites for bearing other modules with stoichiometric precision. The customizable configuration of the functionality on nanodevice is capable of broadening its application scenarios. Moreover, based on the theory of size‐dependent renal filtration for biomacromolecules,^[^ ^10^ ^]^ we speculate that programming the length of the TDF edge enables tunable renal clearance. The second module comprises a Kim‐1‐targeting ligand (an anti‐Kim‐1 peptide)^[^ ^11^ ^]^ enabling nanodevice to stably localize Kim‐1 on the renal tubules. Third is the reporting module consisting of a bioinert fluorophore to offer tracking signal. When visualizing Kim‐1 in situ via real‐time fluorescence imaging, the photostable near‐infrared (NIR) dye (IR 800CW) was selected owing to its weakened light‐tissue interactions, light attenuation, and background autofluorescence within the NIR window.^[^ ^12^ ^]^

To fabricate the nanodevice, the azide‐modified anti‐Kim‐1 peptide and the IR 800CW were separately conjugated with oligonucleotides complementary to the TDF docking strands via a copper‐free click reaction, yielding the Kim‐1‐targeting (DNA‐Pep_Kim‐1_) and NIR signaling modules (DNA‐Rep_800CW_) (Figure S1–S3, Supporting Information).^[^ ^13^ ^]^ Then, we designed three types of TDF scaffolds: TDF_7_, TDF_17_, TDF_37_ with different sizes, in which each edge of the TDF respectively containing 7, 17, or 37 base pairs was about 2.4, 5.8, 12.6 nm. These scaffolds were assembled by annealing the multiple component oligonucleotides (Table S1, Supporting Information),^[^ ^14^ ^]^ which carried four docking strands that were further stoichiometrically hybridized with functional modules, forming integrated nanodevice. After assembly, native polyacrylamide gel electrophoresis (PAGE) was conducted to verify the stepwise assembly of Kim‐TDF (bearing one Kim‐1‐targeting module and three NIR signaling modules). As shown in Figure 1B and Figure S4 (Supporting Information), with the step‐by‐step addition of DNA strands, the gel mobility of the corresponding assemblies gradually declined with the increase in molecular mass, and there clearly appeared a distinct band with the slowest electrophoretic shift in Kim‐TDF lane, suggesting the successful formation of Kim‐TDF. The atomic force microscopy (AFM) and dynamic light scattering (DLS) analysis showed that the hydrodynamic diameters of these structures were ≈4.85, 10.10, and 21.04 nm, respectively (Figure 1A; Figures S5 and S6, Supporting Information). The above data confirmed the successful assembly of Kim‐TDF.

### 2.2 Structural Integrity of the Nanodevices

**Figure 2:**
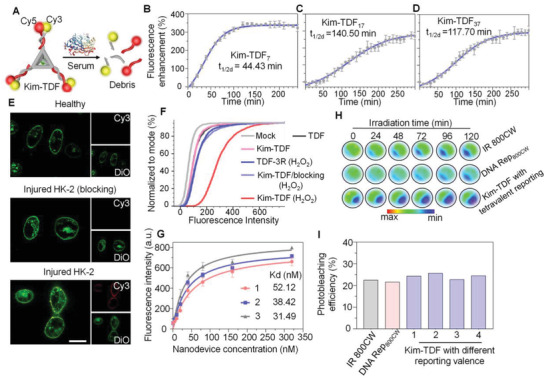
The characterizations of Kim‐TDF nanodevice's functionalities. A) The schematic illustration of degradation of nanodevice. B–D) The degradation kinetics of Kim‐TDF_7_, Kim‐TDF_17_ and Kim‐TDF_37_ via FRET analysis. Data represent mean ± standard deviation (S.D.) (*n* = 3). E–G) The Kim‐1‐binding ability of Kim‐TDF. E) The CLSM images of the HK‐2 cells incubated with Cy3‐labelled nanodevice. Cell membrane were stained with 3,3'‐dioctadecyloxacarbocyanineperchlorate (DiO). The concentration of Kim‐TDF and corresponding TDF‐3R probe was 200 nM. Scale bars: 30 µm. F) FCM analysis of Kim‐1‐binding ability of Cy3‐labelled Kim‐TDF. The concentration of Kim‐TDF and corresponding TDF‐3R probe was 200 nM. G) Analysis of the binding affinity of Kim‐TDF with different targeting valence (1, 2, 3) for HK‐2 cells treated with H_2_O_2_. Data represent mean ± S.D. (*n* = 3). H) NIR fluorescence images of different groups at different scanning intervals (equivalent fluorophore concentration: 200 nM). I) The photobleaching efficiency of Kim‐TDF.

Having fabricated the nanodevices, we evaluated their structural integrity in physiological environments, which is crucial for their in vivo imaging application. The stability of the TDF docking strands against the degradative conditions was studied via fluorescence resonance energy transfer (FRET) analysis. Three pendant fluorescent strands in Kim‐TDF were modified with a pair of donor‐acceptor dyes (Cy3 and Cy5). Then, the resulting Kim‐TDF were incubated with 10% fresh mouse serum for different times at 37 ℃ (**Figure** **2**A). As shown in Figure 2B–D, the FRET efficiency of Kim‐TDF gradually declined owing to the degradation of pendant strands. Compared with double strands DNA (dsDNA) possessing a degradation half‐life (*t*_1/2d_) of 33.09 min (Figure S7, Supporting Information), the *t*_1/2d_ of Kim‐TDF_7_’s overhangs (44.43 min) did not observably increase, suggesting the low resistance to DNase degradation. By contrast, the stability of pendant strands in Kim‐TDF_17_ and Kim‐TDF_37_ under physiological conditions was significantly enhanced as evidenced by the prolonged *t*_1/2d_ of Kim‐TDF_17_ (140.5 min) and Kim‐TDF_37_ (117.7 min), thus prioritizing application in vivo. The improved resistance to enzymatic degradation for Kim‐TDF_17_ and Kim‐TDF_37_ may result from higher structural stability than Kim‐TDF_7._

### 2.3 Targeting of Kim‐1‐Positive Cells

We next tested the Kim‐1‐targeting ability of the nanodevice with in vitro cultured Kim‐1‐positive cells. The Kim‐TDF_17_ which has optimal stability of pendant strands is chosen to conduct cell experiments. The human renal tubular epithelial (HK‐2) cells with overexpressed Kim‐1 (induced by H_2_O_2_ treatment described elsewhere,^[^ ^15^ ^]^ Figure S9, Supporting Information) were incubated with Kim‐TDF (carrying trivalent Cy3‐labelled DNA reporters and monovalent DNA‐Pep_Kim‐1_) and TDF‐3R (just carrying trivalent Cy3‐labelled DNA reporters) for 0.5 h and then analyzed by flow cytometry (FCM). Meanwhile, the H_2_O_2_‐pretreated HK‐2 cells subsequently blocked by Kim‐1 antibody were used as a control to verify the real Kim‐1‐specific targeting behavior of nanodevice. As shown in Figure 2F, the mean fluorescence intensity of the HK‐2 cells treated with Kim‐TDF and TDF‐3R was almost comparable to that of the mock group, suggesting that there was no obvious interaction between DNA nanostructures and untreated HK‐2 cells. Upon pretreatment with H_2_O_2_, the HK‐2 cells incubated with TDF‐3R probes still performed no significant fluorescence change. By contrast, the distinct enhanced fluorescence signals were observed for H_2_O_2_‐stimulated cells incubated with Kim‐TDF, implying the interaction behavior between nanodevice and injured HK‐2 cells. When blocking overexpressed Kim‐1 via Kim‐1 antibody, the fluorescence intensity of injured cells after incubated with Kim‐TDF declined, confirming the Kim‐1‐specific targeting ability of Kim‐TDF. The confocal laser scanning microscopy (CLSM) results further demonstrated the effective interaction between Kim‐TDFs and cytomembrane of injury cells (Figure 2E; Figure S9, Supporting information).

Further, we evaluated the multivalent recognition toward injured HK‐2 cells by modularly assembling targeting valences (from 1 to 3) in Kim‐TDF. As shown in Figure 2G, the equilibrium dissociation constants (*K*_d_) of nanodevices to Kim‐1 gradually reduced along with the increase of targeting valences, implying the targeting valence‐dependent binding affinity of Kim‐TDF to Kim‐1. In addition, 3‐(4, 5‐Dime‐thylthiazol‐2‐yl)‐5‐(3‐carboxymethoxyphenyl)‐2‐(4‐sulfophenyl)‐2H‐tetrazolium (MTT) assay showed no obvious cytotoxicity caused by the nanodevice (Figure S10, Supporting Information). Taken together, these results confirmed the targeting ability of the nanodevice toward Kim‐1‐positive cells.

### 2.4 Validation of NIR Signaling

The photophysical properties of fluorophores are crucial for long‐term temporal tracking of dynamic variation in diseased tissue or image‐guided surgery.^[^ ^16^ ^]^ Thus, we studied the photostability of free IR 800CW, DNA Rep_800CW_, and TDF_17_ scaffold bearing different reporting valences (from 1 to 4) in 1×TAE/Mg^2+^ buffer for long‐term fluorescent imaging. As shown in Figure 2H,I and Figures S11 and S12 (Supporting information), keeping the same concentration of fluorophore (200 nM), upon irradiation for 2 h, the photobleaching efficiency of nanodevices had no obvious decline compared with that of free fluorophore, mainly resulting from that the topological architecture of TDF made the reporting fluorophores mutually independent. And the results also demonstrated that modification and assembly did not influence the photophysical properties of IR 800CW. Notably, continuous irradiation of nanodevice experiment also exhibited the photostability of Kim‐TDF permits long‐term temporal tracking of dynamic variation of Kim‐1 on the renal tubules.

### 2.5 Renal Clearance of TDF Scaffold

With well‐characterized nanodevices, we investigated the pharmacokinetics behaviors of the TDF scaffolds by quantifying the fluorescence intensity of TDF in the blood at a different post‐injection time. The injection dose‐time curves demonstrated that all TDF macroscopically exhibited a two‐compartment feature of in vivo kinetics (Figure S13, Supporting Information).^[^ ^17^ ^]^ And the distribution (*t*_1/2*α*_) and elimination (*t*_1/2*β*_) half‐life gradually prolonged with the increase of scaffolds size, implying that the smaller size of TDF, the faster elimination from the body.

**Figure 3:**
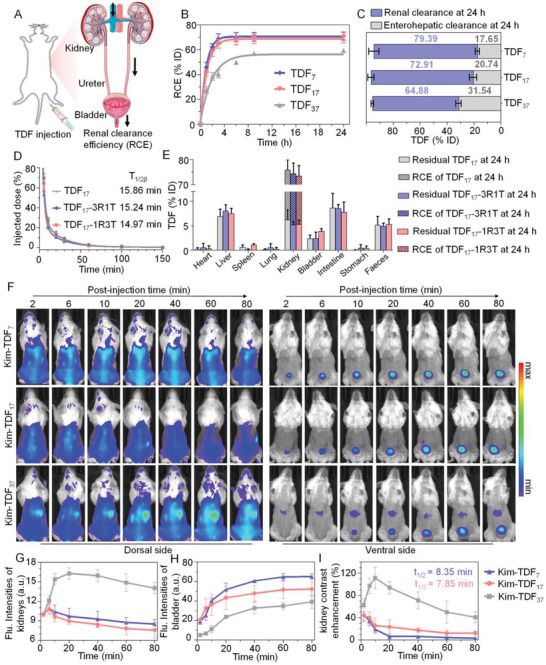
Renal clearance studies of TDFs. A) Schematic illustration of the excretion of TDFs probes through the urinary tract. B) RCE of TDFs at different post‐injection time. Data represent mean ± S.D. (*n* = 4). C) The amount of TDFs excreted from renal pathway (blue bar) and enterohepatic pathway (gray bar) of mice at 24 h post‐injection. Data represent mean ± S.D. (*n* = 4). D) Blood concentration (% ID) decay of nanodevices with different module configurations in healthy mice. Data represent mean ± S.D. (*n* = 4). E) Analysis of the amount of TDFs with different module configurations excreted from kidneys into urine and residual TDFs in major organs of mice after 24 h injection of TDFs. Data represent mean ± S.D. (*n* = 4). F) NIR images of healthy mice at different post‐treatment time points of Kim‐TDFs. G) NIR fluorescence intensities of kidneys and H) bladder at different post‐treatment time points of Kim‐TDFs in healthy mice. Data represent mean ± S.D. (*n* = 3). I) Percentage of kidney contrast enhancement at different post‐treatment time points of Kim‐TDFs in healthy mice. Data represent mean ± S.D. (*n* = 3).

Previous reports had confirmed the kidney‐targeting of TDF scaffold,^[^ ^4^, ^5^, ^18^ ^]^ yet its renal clearance efficiency (RCE) remains unknown (**Figure** **3**A). Therefore, we next determined the RCE of TDF scaffold via quantifying the fluorescence intensity of urine at different times after intravenous injection. The results (Figure 3B) demonstrated that the RCE of TDF_7_ was 62.30% injected doses (ID) at 2 h post‐injection and 70.96% ID at 24 h post‐injection, which was slightly higher than that of TDF_17_ (57.48% ID at 2 h post‐injection and 69.65% ID at 24 h post‐injection). The TDF_37_ with largest size exhibited lowest RCE (38.43% ID at 2 h post‐injection and 59.33% ID at 24 h post‐injection). After being injected for 24 h, major organs of mice were collected and homogenized to analyze the distribution of residuals in the body. As shown in Figure S14 (Supporting information), the DNA residuals were mainly located in the liver, intestine, and feces, and negligible DNA residuals were found in other organs. Moreover, the proportion of enterohepatic clearance for TDF gradually enhanced along with an increase in the size of TDF scaffold (Figure 3C). To further demonstrate that bearing the functional modules has no significant effect on pharmacokinetics of the TDF scaffolds, the nanodevices with different module configurations (TDF‐3R1T: carrying trivalent reporting and monovalent targeting modules; TDF‐1R3T: carrying monovalent reporting and trivalent targeting modules) were intravenously injected into mice, and the vein blood was withdrawn at predetermined time intervals to determine corresponding *t*_1/2*β*_ and RCE. Figure 3D,E showed that these two nanodevices possessed similar *t*_1/2*β*_, RCE, and metabolic distribution compared with TDF_17_ scaffold, implying that TDF scaffolds determine the pharmacokinetics behavior of nanodevices.

### 2.6 Renal Clearance Kinetics of Nanodevice

We further investigated the clearance kinetics of the nanodevices with different TDF scaffolds by longitudinal real‐time fluorescence imaging. To obtain the optimal effect of events tracking, the nanodevice with one Kim‐1‐targeting module and three NIR signaling modules was intravenously injected into living mice. As shown in Figure 3F–I and Figures S15 and S16 (Supporting Information), a fluorescence signal began to appear in the kidneys and bladder as early as 2 min post‐injection of Kim‐TDF_7_ and Kim‐TDF_17_. Then the fluorescence intensity of the kidney reached its maximum at 6 min post‐injection for these two Kim‐TDF, meanwhile, the bladder signals gradually increased after injection. The ratios of bladder‐to‐kidney (BTK) fluorescence intensity for Kim‐TDF_7_ were determined to be ≈2.33 and ≈7.67 at 6 min and 80 min post‐injection which was almost the same as that of Kim‐TDF_17_ (≈2.93 and ≈6.87 at 6 min and 80 min post‐injection), suggesting comparable renal excretion rate for Kim‐TDF_7_ and Kim‐TDF_17_ (Figure S18, Supporting Information). By contrast, the fluorescent peak of kidney appeared at 20 min after injecting Kim‐TDF_37_ (maximum signal of kidney respectively increased 49.68 and 50.51% compared with that treated with Kim‐TDF_7_ and Kim‐TDF_17_), followed by a slow descent. The different fluorescent kinetics between Kim‐TDF was possibly induced by different renal clearance pathways.^[^ ^19^ ^]^ Furthermore, the BTK intensity in mice after injected Kim‐TDF_37_ was lower (≈0.56 and ≈2.92 at 6 min and 80 min post‐injection) than that in other groups, reconfirming the relatively sluggish renal excretion of Kim‐TDF_37_. Notably, compared to the fluorescence intensity of skin, the intensity of kidney in Kim‐TDF_7_ and Kim‐TDF_17_ administrated mice did not observably enhance, whereas the renal signal after being treated with Kim‐TDF_37_ significantly increased.

Quantitative studies on the contrast enhancements demonstrated that the renal contrast enhancement ([(mean intensity of kidney/mean intensity of surrounding tissue)‐1] × 100%) for Kim‐TDF_7_ and Kim‐TDF_17_ treated mice increased to 46.50% and 44.46% at 2 min post‐injection, respectively, then rapidly declined to 3.68% and 12.68% at 80 min following single‐exponential decay kinetics with the decline half‐time (*t*_1/2_) of 8.35 and 7.85 min (Figure 3I). This negligible improvement in kidney contrast mainly resulted from the rapid renal excretion of Kim‐TDF_7_ and Kim‐TDF_17_. However, the kidney images acquired with Kim‐TDF_37_ were different from the others two groups. Upon treatment with Kim‐TDF_37_, the renal contrast enhancement rapidly reached 62.56% at 2 min post‐injection, then gradually increased to maximum values (111.36%) at 20 min post‐injection, which respectively were 9.38‐ and 5.89‐times higher than the groups treated with Kim‐TDF_7_ (11.87%) and Kim‐TDF_17_ (18.91%). The higher kidney contrast in healthy mice possibly covered up the Kim‐1‐specific signals in AKI mice, making the fidelity of Kim‐1 imaging unsatisfactory and interfering the early diagnosis of AKI. In addition, upon treatment with Kim‐TDF, the cells with the well‐defined cytoplasm and nuclei are observed from hematoxylin and eosin staining (H&E) of the major organs, suggesting the negligible toxicity of Kim‐TDF to mice (Figure S19, Supporting information).

### 2.7 In Vivo Renal Clearance Pathways of Nanodevice

**Figure 4:**
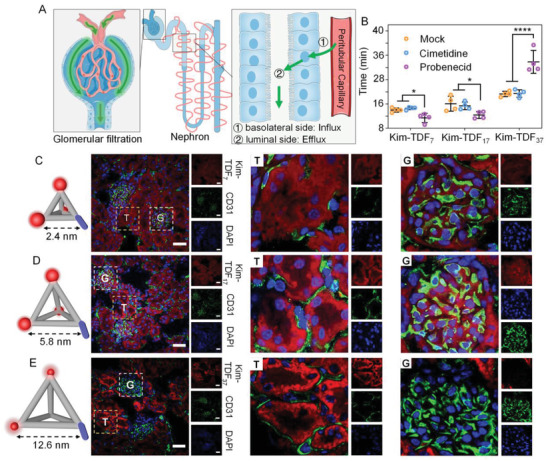
In vivo renal clearance pathways of Kim‐TDFs in health mice. A) Schematic illustration of two distinct renal clearance pathways in the kidneys. B) The *t*_1/2*β*_ of Kim‐TDFs in living mice treated with probenecid or cimetidine. Data represent mean ± S.D. (*n* = 4). Statistical analysis: *p < 0.05, ****p < 0.0001. C,D,E) Fluorescence images of glomerulus and tubules at tissue level at 6 min post‐injection of Cy3‐labelled Kim‐TDFs (red signal). Nuclei were stained with 2‐(4‐amidinophenyl)‐1H‐indole‐6‐carboxamidine (DAPI; blue). Blood vessel stained with anti‐CD31 antibody (green). “G” denotes glomeruli and “T” denotes renal tubular.

In general, there are two main renal clearance pathways including glomerular filtration and renal tubular secretion (**Figure** **4**A).^[^ ^17^, ^20^ ^]^ Owing to the ultra‐microstructure of glomerular at the nanoscale, the glomerular filtration performs a size‐dependent feature that only nanoparticles or molecules smaller than 6 nm in hydrodynamic diameter (or lower than 40 kDa in molecular weight) can passively pass through the glomerular filtration barrier.^[^ ^10^, ^21^ ^]^ On the other hand, the secretion behaviors for renal tubular cells from peritubular capillaries into the lumen of the proximal tubules were regulated by the OAT and the organic cation transporters (OCT) on the basolateral side of proximal tubular cells.^[^ ^22^ ^]^ To deepen the understanding of renal excretion of Kim‐TDF, the renal clearance pathways were studied via measuring the *t*_1/2*β*_ of Kim‐TDF in probenecid (OAT inhibitor)‐pretreated and cimetidine (OCT inhibitor)‐pretreated mice.^[^ ^23^ ^]^ Compared with the results in mock groups, the *t*_1/2*β*_ of Kim‐TDF_7_ and Kim‐TDF_17_ in cimetidine‐blocked mice both exhibited insignificant variation. Surprisingly, Kim‐TDF_7_ and Kim‐TDF_17_ were more rapidly eliminated in probenecid‐blocked mice, as evidenced by the shortened *t*_1/2*β*_, suggesting that the renal clearance of Kim‐TDF_7_ and Kim‐TDF_17_ was possibly associated with tubular secretion (Figure 4B; Figure S20, Supporting information). In contrast, Kim‐TDF_37_ exhibited prolonged *t*_1/2*β*_ in probenecid‐pretreated mice, implying the OAT‐mediated active tubular secretion behavior.

To further confirm the renal clearance pathways of Kim‐TDF, its distribution in the kidneys at 6 min, 1 h, and 4 h post‐injection was investigated by fluorescence microscopy imaging (Figure 4C; Figure S21, Supporting information). As shown in Figure 4C, at 6 min post‐injection, the fluorescence signals of Kim‐TDF_7_ were mainly located in glomeruli and proximal tubules, suggesting the synergistic renal clearance pathways of glomerular filtration and renal tubular secretion for Kim‐TDF_7_. The previous report revealed that the excretion speed of glomerular filtration is faster than renal tubular secretion,^[^ ^19^ ^]^ thus we speculated that Kim‐TDF_7_ were principally eliminated via glomerular filtration, resulting in the slight shortened *t*_1/2*β*_ of Kim‐TDF_7_ in OAT‐blocked mice (Figure 4B; Figure S20, Supporting information). Additionally, authentic information was hardly obtained from the fluorescence images of kidneys at 1 h and 4 h post‐injection owing to the rapid degradation of Kim‐TDF_7_. Compared with Kim‐TDF_7_, Kim‐TDF_17_ showed similar distribution at 6 min post‐injection, implying the similar renal clearance pathways. And only weak fluorescence signals in renal tubulars were observed at 1 h post‐injection, reconfirming the rapid renal clearance of Kim‐TDF_17_ (Figure S21, Supporting information). Interestingly, Kim‐TDF_37_ performed different phenomena that the fluorescence signals were only located in proximal tubules at 6 min post‐injection, reconfirming its OAT‐dependent active tubular secretion pathway.

### 2.8 Diagnosis of AKI In Vivo by NIR Imaging

Next, we demonstrated the diagnosis of early‐stage AKI in living mice by NIR in vivo imaging based on the nanodevice. We first established the murine model of rhabdomyolysis‐induced AKI by intramuscular injection of 50% glycerol (5 mL kg^‐1^) into dehydrated healthy mice.^[^ ^24^ ^]^ The serum creatinine (sCr) and blood urea nitrogen (BUN) levels at different time points post modeling (0, 6, 12, 24, and 36 h post glycerol injection) were analyzed, and the first statistically significant increase in sCr and BUN was observed 36 h post‐induction, which was 6.6‐fold and 5.2‐fold higher than the group at 0 h post‐induction, implying that the earliest time points for sCr or BUN to detect the occurrence of AKI was in the interval of 24–36 h after induced by 50% glycerol (Figure S22, Supporting Information). Moreover, immunofluorescence staining of Kim‐1 in kidneys at different time points post‐treatment was analyzed by immunofluorescence staining, and revealed that a significant increase in Kim‐1 expression was observed as early as 12 h post modeling, which maintained a high level after 24 h (Figure S23, Supporting Information). In addition, the H&E staining showed that the abnormal tubular morphology was first observed after 12 h induced by 50% glycerol (Figure S24, Supporting Information). These results confirmed that the AKI models were successfully built and that Kim‐1 indeed can serve as the biomarkers for early‐stage AKI.

**Figure 5:**
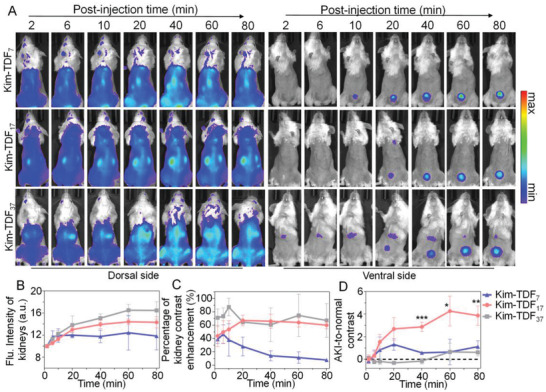
Dependency of AKI‐to‐normal contrast on the TDF size. A) NIR images of living mice after injected with Kim‐TDFs at 12 h post‐induction with 50% glycerol. B) NIR fluorescence intensities of kidneys at different post‐injection time points of Kim‐TDFs. C) The percentage of kidney contrast enhancement at different post‐injection time points of Kim‐TDFs in living mice at 12 h post‐induction with 50% glycerol. D) AKI‐to‐normal contrast at different post‐injection time points of Kim‐TDFs in living mice at 12 h post‐induction with 50% glycerol. Statistical analysis in (D) Kim‐TDF_17_ versus other groups; mean ±  S.D. (*n* = 3); *p < 0.05, **p < 0.01, ***p < 0.001.

Next, the AKI model mice were intravenously injected with the nanodevices for in vivo NIR imaging. We investigated the dependency of the AKI‐to‐normal contrast on the size of the nanodevices by intravenously injecting Kim‐TDFs into the 12 h glycerol‐post‐induced mice which overexpressed Kim‐1. As shown in **Figure** **5**A,B and Figures S25–S27 (Supporting Information), the variation trends of kidney fluorescence after injected with Kim‐TDFs were similar, but the maximum of kidney signal gradually enhanced with the increase of Kim‐TDFs size. Meanwhile, the bladder fluorescence and BTK ratios in Kim‐TDF_7_ groups at 10 min and 80 min post‐injection were both higher than that treated with Kim‐TDF_17_ and Kim‐TDF_37_, possibly owing to the shorter degradation half‐life of Kim‐TDF_7_ (Figure S28, Supporting information), thus resulting in the inferior kidney contrast (Figure 5C). The AKI‐to‐normal contrast at 12 h post‐induced by 50% glycerol was defined as [mean of renal contrast enhancement (12 h post‐induction)/mean of renal contrast enhancement (0 h post‐induction)‐1]. The AKI‐to‐normal contrast for using Kim‐TDF_7_ probes was about 1.31 (at 20 min post‐injection). For Kim‐TDF_37_ probes, high renal contrast was observed in both, mice at 0 h and 12 h post‐induction, so its AKI‐to‐normal contrast approached 0 within the NIR imaging time (Figure 5D), demonstrating that the Kim‐TDF_37_ probes had inferior authenticity to visualize Kim‐1. By contrast, the AKI‐to‐normal contrast for using the Kim‐TDF_17_ gradually increased along with time and reached the maximum value (≈4.27) at 60 min post‐injection, which was 3.26‐fold higher than group of Kim‐TDF_7_. Taken together, we can conclude that compared to the nanodevices of other sizes (Kim‐TDF_7_ and Kim‐TDF_37_), Kim‐TDF_17_ possessing the most biological stability can be more rapidly cleared from healthy kidneys, resulting in lower renal retention, which further enhanced the AKI‐to‐normal contrast.

**Figure 6:**
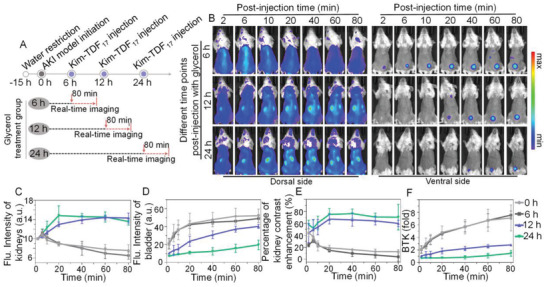
Real‐time in vivo NIR imaging of AKI mice. A) Schematic illustration of glycerol‐pretreated (5 mL kg^−1^) mice and NIR imaging at different post‐treatment time points. B) NIR images of living mice after injected with Kim‐TDF_17_ at different post‐induction time points with 50% glycerol. C) NIR fluorescence intensities of kidneys and D) bladder at different post‐injection time points of Kim‐TDF_17_ in living mice at 6, 12, 24 h post‐induction with 50% glycerol. E) Percentage of kidney contrast enhancement and F) the ratios of BTK intensity at different post‐injection time points of Kim‐TDF_17_ in living mice at 6, 12, 24 h post‐induction with 50% glycerol. (C–F) Data represent mean ±  S.D. (*n* = 3).

Having obtained the nanodevice with the optimal AKI‐to‐normal contrast, we finally evaluated the timeliness of this approach in AKI early diagnosis. We employed Kim‐TDF_17_ to diagnose the mice at different time points post AKI induction (6, 12, and 24 h, respectively, shown in **Figure** **6**A). At 6 h post modeling, the variation tendency of Kim‐TDF_17_ signals in kidneys and bladder was almost consistent with that of the healthy group (0 h), suggesting that the histopathologic renal damage at 6 h post modeling was too slight to influence the pharmacokinetics of Kim‐TDF_17_ (Figure 6B; Figures S29 and S30, Supporting Information). Nevertheless, at 12 h post modeling, the renal signals gradually enhanced, and the signal appearance in the bladder delayed to 20 min post‐injection of Kim‐TDF_17_. Meanwhile, the increase amplitude of bladder signals was lower than that of mice after 0 h and 6 h pre‐treatment of 50% glycerol (Figure 6B,D). Its ratios of BTK signals were demonstrated to be ≈1.27 and ≈2.81 after systemically administrated with Kim‐TDF_17_ at 6 min and 80 min, which were respectively 2.10‐fold and 2.69‐fold lower compared with the control group at 0 h post‐treatment (Figure 6F). Especially, the renal contrast enhancement of the mice after 12 h treatment with 50% glycerol gradually increased within 20 min injection, and then tardily declined from ≈67.07% at 20 min post‐injection to 59.90% at 80 min post‐injection, which was dramatically higher than that of mice at 0 h post‐treatment (Figure 6E). Note that the variation trends of TDF_17_‐3R (only carrying trivalent reporting ligands) signals at 12 h post‐treatment with 50% glycerol were roughly similar with that of Kim‐TDF_17_ signals in healthy mice (0 h), suggesting the real Kim‐1‐specific targeting behavior of nanodevice (Figures S32 and S33, Supporting Information). In addition, the higher renal contrast enhancement at 24 h post‐induced mice after injected Kim‐TDF_17_ was observed owing to the synergistic effect of targeting ability and prolonged *t*_1/2*β*_ of Kim‐TDF_17_ (Figure S29, Supporting Information)_._ These results indicate that Kim‐TDF_17_ exhibits significantly higher accumulation in AKI kidneys (12 h post AKI induction) compared to that in healthy ones, which can be attributed to its ability to target Kim‐1 molecules overexpressed on the renal tubule cytomembranes of AKI kidneys, thus empowers the nanodevice with the specificity to AKI (high AKI‐to‐normal contrast).

### 2.9 Kim‐TDF‐Based Urinalysis

**Figure 7:**
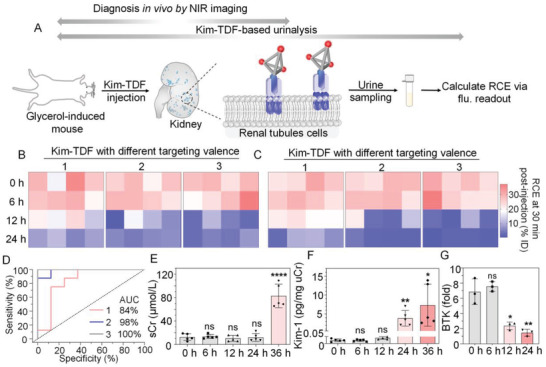
Diagnosis of glycerol‐induced AKI in living mice. A) The schematic illustration of diagnosis in vivo by NIR imaging and Kim‐TDF‐based urinalysis. B) Kim‐TDF‐based urinalysis of living mice after injected by Kim‐TDF with different valence (1, 2, 3) of targeting at different time points post‐induction with 50% glycerol. (The reporting fluorophore in Kim‐TDF was IR 800CW). C) Kim‐TDF‐based urinalysis of living mice after injected by Kim‐TDF with different valence (1, 2, 3) of targeting at different time points post‐induction with 50% glycerol. (The reporting fluorophore in Kim‐TDF was FAM). D) Receiver operating characteristic analysis for living mice after injected by Kim‐TDF with different valence (1, 2, 3) of targeting at 12 points post‐induction with 50% glycerol. E,F) Change in sCr, urinary Kim‐1 in living mice at different time points post‐induction with 50% glycerol. Data represent mean ±  S.D. (*n* = 5). G) The BTK ratios of fluorescence intensity in living mice after injected by Kim‐TDF at different time points post‐induction with 50% glycerol. Data represent mean ±  S.D. (*n* = 3). Statistical analysis in (E–G) 0 h versus other groups; *p < 0.05, **p < 0.01, ****p < 0.0001; ns, no significance.

To evaluate the translational potential of Kim‐TDF, glycerol‐induced mice were directly detected via collecting urine and calculating the RCE of nanodevice at 30 min post‐injection with nanodevice (**Figure** **7**A). Owing to the targeting valence‐dependence of binding affinity of nanodevices to Kim‐1, the RCE of Kim‐TDF assembling multiple targeting valences (from 1 to 3) was investigated to screen out optimal Kim‐TDF with the best urinalysis performance. As shown in Figure 7B, for Kim‐TDF probe with monovalent targeting, the first statistically significant decline of RCE was observed at 12 h post modeling, which was consistent with the in vivo diagnosis by real‐time NIR imaging. Moreover, with the increase of the targeting valence in nanodevice, the accuracy of urinalysis for 12 h post modeling enhanced, as evidenced by the increased area under curve (AUC) (Figure 7D). Note that when reporting module (Rep_800CW_) in Kim‐TDF was replaced with fluorescein (FAM)‐labeled DNA, the sensitivity and accuracy of urinalysis were unaffected (Figure 7C). This reporting module‐independence permits Kim‐TDF‐based urinalysis to be more promising than diagnosis via real‐time NIR imaging.

We compared the timeliness of our approach with the conventional in vitro detection methods, including the blood test of the biomarkers sCr and BUN, and the urinalysis of Kim‐1. Based on our research above, the earliest time points for sCr or BUN to detect the occurrence of AKI was in the interval of 24–36 h after induced by 50% glycerol (Figure 7E; Figure S22, Supporting information). Further, the marker urinary Kim‐1 exhibited more sensitive than sCr or BUN, as evidenced by its first statistically significant increases at 24 h (162.33‐fold) post‐induced by 50% glycerol (Figure 7F). Note that the ratio of BTK intensity in living mice performed the first statistically significant decrease at 12 h (0.35‐fold) post‐treatment with 50% glycerol, which coincided with Kim‐TDF‐based urinalysis and was earlier than the sCr‐, BUN‐based blood test and Kim‐1‐based urinalysis (Figure 7G). Overall, we can conclude that Kim‐TDF‐based NIR imaging and urinalysis enable the diagnosis of AKI onset hours earlier than that can be detected by the conventional in vitro assays.

## 3 Conclusion

In this study, the programmable TDF nanoplatform integrating multiple functionalities including kidney‐targeting module, NIR fluorescence module, and Kim‐1‐targeting module allows real‐time tracking of the dynamic variation of in situ Kim‐1, which can be used to diagnose AKI at the early stage. With the ongoing pandemic of coronavirus disease 2019 (COVID‐19), a non‐trivial number of severe/critical COVID‐19 patients suffer AKI concurrently induced by the illness,^[^ ^25^ ^]^ which makes early diagnosis of AKI particularly important. Though many reports demonstrated that urinary Kim‐1 outperformed other AKI markers in preclinical biomarker qualification studies,^[^ ^7a,b^ ^]^ the variation of urinary Kim‐1 did not synchronize with histopathologic damages since the time from the expression of Kim‐1 to the exfoliation of ectodomain and further transportation toward bladder is non‐negligible.^[^ ^26^ ^]^ Thus, direct detection of Kim‐1 on renal tubular theoretically permits diagnosis of the onset of AKI earlier than Kim‐1‐based urinalysis. Nevertheless, the principal limitation in the visualization of in situ Kim‐1 with high signal‐to‐background contrast is the lack of an imaging probe that enables customization adapting for renal microenvironment. Compared to traditional probes,^[^ ^26a^ ^]^ our TDF‐based nanodevice shows several unprecedented advantages.

First, the modularized nanodevice enables the assembly of functional modules with stoichiometric precision. This customizable ability dispenses with many chemical coupling processes which not only take superabundant labor forces and times but possibly damage the intrinsic ability of each module. Notably, as a loadable carrier, the TDF scaffold possessing inherent kidney‐targeting enables assimilation of the undesiring pharmacokinetics of functional modules. For example, upon hybridization with TDF scaffold, the liver‐accumulating pharmacokinetics of fluorophore (IR 800CW)^[^ ^27^ ^]^ turned into renal‐clearable behavior (Figure 3). Most importantly, depending on the application scenarios, the TDF nanodevice is capable of magnifying tracking signal or binding affinity by bearing multivalent reporting or targeting modules to acquire optimal performance. For example, when visualizing Kim‐1 via real‐time NIR imaging, the photophysical properties of nanodevice are crucial for long‐term temporal tracking of AKI, thus the number of reporting modules on TDF scaffold should be as more as possible. By contrast, using Kim‐TDF‐based urinalysis for early diagnosis of AKI, of which performance exhibits reporting module‐independence, should maintain the configuration of multivalent targeting to acquire the highest accuracy.

Second, the programmable nanodevice permits customization of size via DNA nanotechnology, and thus empowers nanoprobe with tunable renal clearance pathway. Such unique property of TDF nanodevice offers a new perspective to solute theranostic challenges in renal disease. For example, in our study, the glomerular‐clearable nanodevice was preferentially used for visualization of Kim‐1 because it could be rapidly excreted in healthy mice, minimizing the output crosstalk between passive kidney targeting and ligand‐receptor‐mediated active targeting, thus enhancing AKI‐to‐normal contrast. In addition, renal tubular secretion‐clearable TDF nanodevice has the potential to specifically image renal cell carcinoma.^[^ ^19^ ^]^ Third, the excellent biocompatibility of nucleic acid‐based materials greatly weakens the hepatobiliary metabolism‐dependent toxicity, making our TDF nanodevice promising in clinical transformation.

In summary, we developed a TDF nanodevice with customizable functionality and tunable sizes, which allows real‐time tracking of the dynamic variation of Kim‐1 in the kidney of living mice undergoing nephrotoxic exposure. Such an in situ imaging mechanism allows nanodevice to be used as an imaging agent and urinalysis tracers to non‐invasively diagnose the onset of AKI at least 6 h earlier than Kim‐1 urinalysis, at 12 h earlier than sCr and BUN further underlines their potential in clinical translation. We thus envision that this nanodevice may provide implications for the design of smart kidney injury theragnostic tools.

## 4 Experimental Section

### Materials and Characterization

All DNAs were purchased from Sangon Biotech (Shanghai) Co., Ltd. All other reagents and reagent‐grade solvents were purchased from Sigma‐Aldrich unless otherwise stated. Kim‐1 antibody (3809‐2003) was purchased from Bio‐Techne China Co. Ltd. The 3,3′‐dioctadecyloxacarbocyanine perchlorate (DiO) was purchased from Beyotime Biotechnology. Anti‐Kim‐1 peptide was purchased from Nanjing ChenPeptide Biotech. Azide‐modified IR 800CW was purchased from LI‐COR Co., Ltd. UV–vis absorption measurements were performed with a UV‐1800 (Shimadzu corporation). Fluorescence spectra were measured by a FluoroMax‐4 (HORIBA Ltd.). AFM images were obtained using MultiMode 8 AFM (Bruker Ltd.). DLS measurements were acquired on a Zetasizer Nano ZS (Malvern Instruments Ltd.). Fluorescence imaging was performed on an IVIS spectrum imaging system (PerkinElmer, Inc, USA).

### Synthesis of DNA‐Pep_Kim‐1_

Generally, the dimethyl sulfoxide solution (DMSO) of dibenzocyclooctyl‐modified DNA (DBCO‐DNA) (360 nmol) were mixed with Anti‐Kim‐1 peptide (500 nmol) which was dissolved in DMSO. After incubating 24 h at 50 ℃ with shaking, the solution was dialyzed to remove the DMSO. The excess anti‐Kim‐1 peptide was removed by centrifuging at 15 000 rpm. The amount of DNA‐PepKim‐1 conjugation was determined by UV–vis spectrophotometer at 260 nm. The denaturing PAGE (10%) gel electrophoresis is used to characterize DNA‐Pep_Kim‐1_ (the loading mass of DNA‐Pep_Kim‐1_ is about 0.1 OD (≈3.3 µg)).

### Synthesis of DNA‐Rep_800CW_

The water solution of DBCO‐DNA (360 nmol) was mixed with azide‐modified IR 800CW (500 nmol) which was dissolved in water. After incubating for 24 h at 50 ℃ with shaking, ethanol was added into the reaction system, followed by placing at −80 ℃ for 12 h. Then the samples were centrifuged at 15 000 rpm, and the blue precipitates were collected. The amount of DNA‐Rep800CW conjugation was determined by UV–vis spectrophotometer at 260 nm. The denaturing PAGE (10%) gel electrophoresis is used to characterize DNA‐Rep_800CW_ (the loading mass of DNA‐Rep_800CW_ is about 0.1 OD (≈3.3 µg)).

### Synthesis of Engineering Kim‐TDF

First, all component strands with the sequences listed in Table S1 (Supporting Information) were assembled at equimolar ratio, and the final concentration of each strand was 5 µM. All component strands were combined in 1 × TAE/Mg^2+^ buffer (40 mM Tris, 2 mM EDTA•2Na•2H_2_O, 20 mM acetic acid, 12.5 mM (CH_3_COO)_2_Mg•4H_2_O, pH = 8.2, adjusted by acetic acid). The solution was heated to 95 ℃ for 5 min and then quickly cooled down to 4 ℃. Then the DNA‐Rep_800CW_ and DNA‐Pep_Kim‐1_ were added to the resulting solution with a corresponding stoichiometric ratio, followed by heating to 40 ℃ for 5 min and then slowly cooling down to room temperature to form the desired nanostructure.

### Characterization of Kim‐TDF

1. PAGE analysis. 40% acrylamide (19:1, acrylamide/bisacrylamide) solution was added in 1 × TAE/Mg^2+^ buffer, 75 µL ammonium persulfate and 7.5 µL tetramethyl ethylenediamine (TEMED) were used as initiating agent and accelerator separately. Then the sample was mixed with an equivalent amount of loading dye and characterized at 4 ℃ (100 V, constant voltage) in 1 × TAE/Mg^2+^ buffer. After electrophoresis, the gels were immersed into gelred for staining, visualized by UV illumination (254 nm), and photographed by a digital camera. 2. AFM imaging. A drop of 3 µL sample solution was spotted onto freshly cleaved mica surface (Electron Microscopy Sciences) and incubated for 10 s to allow samples to absorb onto the substrate. Then the sample drop was washed off by 30 µL magnesium acetate solution (2 mM) and dried by compressed air. The nanogels were imaged in the air in tapping mode on a FM‐Nanoview 1000 AFM.

### Degradation Kinetics of Kim‐TDF

Three pendant fluorescent strands in Kim‐TDF were modified with a pair of donor‐acceptor dyes (Cy3 and Cy5). The resulting Kim‐TDF probes (20 nM) were incubated with 10% (v/v) fresh mouse serum for different times at 37 ℃. And the fluorescence intensity (excitation: 550 nm) was continuously measured in a microplate reader at a wavelength of 620 and 670 nm.

### Cell Culture

Human kidney 2 (HK‐2) cells purchased from China Type Culture Collection (CTCC) and cultured in Dulbecco's modified Eagle's medium (DMEM, Gibco) containing 10% fetal bovine serum (FBS, Gibco) and 1% penicillin/streptomycin (Gibco) at 37 ℃ in a humidified atmosphere with 5% CO_2_.

### In Vitro Cytotoxicity Study of Kim‐TDF

The HK‐2 cells were seeded in the 96‐well plates with the density of 1 × 10^4^ cells per well and followingly cultured for 24 h. Then, the cells were incubated with different concentration of Kim‐TDF at 37 ℃ for 48 h. 20 µL of MTT solution (5 mg mL^−1^) was added to each well and the plates were incubated for 4 h. And then the MTT formazan crystals were dissolved with 250 µL DMSO at room temperature. Finally, the absorbance was measured in a microplate reader at a wavelength of 490 nm.

### Kim‐1 Targeting Ability

To investigate the Kim‐1‐targeted ability of Kim‐TDF, HK‐2 cells were seeded into 12‐well plates and incubated overnight. After being pretreated with H_2_O_2_ (300 µM) for 4 h, HK‐2 cells were treated with TDF or Kim‐TDF at a concentration of 0.2 µM for 0.5 h. After removing the culture medium and washing with PBS, the cells were incubated with Opti‐MEM medium containing DiO at 37 ℃ for incubating 0.5 h. Then the medium was replaced with the fresh one and the staining was examined using CLSM (Zeiss LSM 510 META, Carl Zeiss, Germany) and flow cytometry (LSRFortessa, Becton Dickinson). H_2_O_2_‐stimulated cells with blocking treatment of Kim‐1 antibody were also tested as control.

### Binding Affinity of Kim‐TDF with Different Targeting Valence

HK‐2 cells were seeded into 12‐well plates and incubated overnight. After being pretreated with H_2_O_2_ (300 µM) for 4 h, HK‐2 cells were treated with Kim‐TDF (with different targeting valence) at a different concentration for 0.5 h. Then the medium was replaced with the fresh one and examined by flow cytometry (LSRFortessa, Becton Dickinson). The binding affinity (*K*_d_) of Kim‐TDF was evaluated by fitting the dependence of fluorescence intensity (Y) and the concentrations of aptamer (X) into the one‐site saturation equation Y = Bmax X/(*K*_d_ + X), using GraphPad Prism 7.0

### Photostability of Kim‐TDF

Free IR 800CW, DNA Rep_800CW_, and TDF_17_ scaffold bearing different reporting valences (from 1 to 4) in 1 × TAE/Mg^2+^ buffer with the same concentration of fluorophore (200 nM) were added into 96‐well plates and irradiated for 2 h. Fluorescence images were acquired using the IVIS spectrum imaging system with excitation at 745 nm and emission at 790 nm. The photostability of Kim‐TDF was studied by analyzing NIR fluorescence intensities.

### Animal Experiments

The male ICR mice (4‐week‐old) were purchased from Chinese Academy of Sciences (Shanghai, China). All animal studies in vivo were performed in accordance with National Institutes of Health animal care guidelines and use of laboratory animals proved by the Animal Ethics Committee of Shanghai Jiao Tong University (A2019104).

### Pharmacokinetic Studies

ICR mice were randomly selected and treated with saline (0.2 mL), probenecid (intravenous injection, 150 mg kg^–1^ body weight) or cimetidine (intravenous injection, 150 mg kg^–1^ body weight) 30 min prior to Kim‐TDF (intravenous injection). For glycerin‐treated groups, mice were treated with glycerin followed by injection of Kim‐TDF at 6 h, 12 h, or 24 h post‐treatment of glycerin. At predetermined time intervals, orbital vein blood (20 µL) was withdrawn and the fluorescence intensity of blood was determined by microplate reader.

### Renal Clearance Studies

Mice were intravenously injected with Kim‐TDFs, and placed in metabolic cages. Urine was collected at 1, 2, 3, 6, 9, and 24 h post‐injection, diluted in PBS and centrifuged at 4500 rpm for 10 min and filtered by 0.22 µm syringe filter, then was quantified using fluorescence spectra. At 24 h post‐injection, mice were sacrificed and major organs were collected, homogenized in PBS buffer (10 mM, pH 7.4) and centrifuged at 4500 rpm for 15 min to remove insoluble components. The supernatant containing extracted molecules was taken for fluorescence spectra analysis.

### Real‐Time In Vivo NIR Fluorescence Imaging in Living Mice

Mice were treated with glycerin followed by intravenous injection of Kim‐TDFs at 6, 12, or 24 h post‐treatment of glycerin. Real‐time NIR fluorescence imaging was conducted for 80 min after intravenous injection of Kim‐TDFs in living mice treated with glycerin at t = 0, 6, 12, or 24 h post‐treatment. Fluorescence images of Kim‐TDFs were acquired using the IVIS spectrum imaging system with excitation at 745 nm and emission at 790 nm. NIR fluorescence intensities of kidneys in living mice were analyzed by the ROI analysis.

### Quantification of sCr, BUN, Urinary Kim‐1 Assay

Blood was collected from the orbit in living mice under anesthesia at t = 0, 6, 12, 24, or 36 h post‐treatment of glycerin. The collected blood samples were centrifuged for 15 min at 4500 rpm. Urine was collected by metabolic cages at t = 0, 6, 12, 24, or 36 h post‐treatment of glycerin, then were centrifuged for 15 min at 4500 rpm, and filtered by 0.22 µm syringe filter. sCr, BUN, urinary Kim‐1 were determined using commercial kits according to the manufacturer′s protocol.

### Statistical Analysis

The results presented are representative of at least three independent experiments and data were shown as mean ± standard deviation (S.D.).The student's t‐test (two‐tailed, unpaired) was used to determine the statistical significance (GraphPad Prism 8.0). *p* < 0.05 was considered statistically significant.

## Conflict of Interest

The authors declare no conflict of interest.

## Supporting information

Supporting information

## Data Availability

The data that support the findings of this study are available from the corresponding author upon reasonable request.
