# Supplementary material for: Molecular Visualization of Early‐Stage Acute Kidney Injury with a DNA Framework Nanodevice
Source: Adv Sci (Weinh). 2022 May 4;9(20):2105947. doi: 10.1002/advs.202105947 (PMC9284180; doi:10.1002/advs.202105947)
Supplement: Supplementary file 1 — Supporting information [file ADVS-9-2105947-s001.pdf]

ADVANCED SCIENCE
Open Access

Supporting Information

for *Adv. Sci.*, DOI 10.1002/advs.202105947

Molecular Visualization of Early-Stage Acute Kidney Injury with a DNA Framework Nanodevice

Fei Ding, Shuangye Zhang, Suyu Liu, Jing Feng, Jiang Li, Qian Li, Zhilei Ge, Xiaolei Zuo, Chunhai Fan* and Qiang Xia**

Supplementary Information

Molecular Visualization of Early-stage Acute Kidney Injury with a DNA Framework Nanodevice

Fei Ding, Shuangye Zhang, Suyu Liu, Jing Feng, Jiang Li, Qian Li, Zhilei Ge, Xiaolei Zuo, Chunhai Fan*, Qiang Xia**

F. Ding, X. Zuo, Q. Xia
Institute of Molecular Medicine, Department of Liver Surgery
Shanghai Key Laboratory for Nucleic Acid Chemistry and Nanomedicine
Renji Hospital
School of Medicine, Shanghai Jiao Tong University
Shanghai 200127, China
E-mail: xiaqiang@shsmu.edu.cn
F. Ding, S. Zhang, Q. Li, Z. Ge, X. Zuo, C. Fan
School of Chemistry and Chemical Engineering, Frontiers Science Center for Transformative Molecules and National Center for Translational Medicine
Shanghai Jiao Tong University
Shanghai 200240, China
E-mail: fanchunhai@sjtu.edu.cn
S. Liu, J. Feng
Southern Medical University Affiliated Fengxian Hospital
The Second Affiliated Hospital of the Chinese University of Hong Kong (Shenzhen)
Shenzhen 518172, China
E-mail: fengjing71921@163.com
J. Li
Bioimaging Center, Shanghai Synchrotron Radiation Facility
Zhangjiang Laboratory
Shanghai Advanced Research Institute, Chinese Academy of Sciences

Shanghai 201210, China

Q. Li

WLA Laboratories

Shanghai 201203, China

Table S1. The sequence of DNA.

DNA	Sequence (5'-3')
Kim-TDF_7	
A_7	**TTG ACC TGT GAA** GAG CGT TAG CCA CAC ACA CAG TC
B_7	**TTG ACC TGT GAA** TTA GGC GAG TGT GGC AGA GGT GT
C_7	**TTT TTT TTT TTT TTT TTT TT** CGC CTA AAC AAG TGG AGA CTG TG
D_7	**TTG ACC TGT GAA** AAC GCT CAC CAC TTG AAC ACC TC
Kim-TDF_7 for FRET assay	
A_7-Cy5	Cy5- **TTG ACC TGT GAA** GAG CGT TAG CCA CAC ACA CAG TC
B_7-Cy5	Cy5- **TTG ACC TGT GAA** TTA GGC GAG TGT GGC AGA GGT GT
D_7-Cy5	Cy5- **TTG ACC TGT GAA** AAC GCT CAC CAC TTG AAC ACC TC
Kim-TDF_{17}	
A_{17}	**TTG ACC TGT GAA** ACA TTC CTA AGT CTG AAA CAT TAC AGC TTG CTA CAC GAG AAG AGC CGC CAT AGT A
B_{17}	**TTG ACC TGT GAA** TAT CAC CAG GCA GTT GAC AGT GTA GCA AGC TGT AAT AGA TGC GAG GGT CCA ATA C
C_{17}	**TTT TTT TTT TTT TTT TTT TT**T CAA CTG CCT GGT GAT AAA ACG ACA CTA CGT GGG AAT CTA CTA TGG CGG CTC TTC
D_{17}	**TTG ACC TGT GAA** TTC AGA CTT AGG AAT GTG CTT CCC ACG TAG TGT CGT TTG TAT TGG ACC CTC GCA T
Kim-TDF_{17} for FRET assay	
A_{17}-Cy5	Cy5- **TTG ACC TGT GAA** ACA TTC CTA AGT CTG AAA CAT TAC AGC TTG CTA CAC GAG AAG AGC CGC CAT AGT A
B_{17}-Cy5	Cy5- **TTG ACC TGT GAA** TAT CAC CAG GCA GTT GAC AGT GTA GCA AGC TGT AAT AGA TGC GAG GGT CCA ATA C
D_{17}-Cy5	Cy5- **TTG ACC TGT GAA** TTC AGA CTT AGG AAT GTG CTT CCC ACG TAG TGT CGT TTG TAT TGG ACC CTC GCA T
Kim-TDF_{37}	
$A1_{37}$	**TTG ACC TGT GAA** C CCT GTA CTG GCT AGG AAT TCA CGT TTT AAT CTG GGC TTT GGG TTA AGA AAC TCC CCG
$A2_{37}$	CGC TGG AGG CGC ATC ACC G TTT GC GTA TGT GTT CTG TGC GGC CTG CCGTCC CGT GTG GG
$B1_{37}$	**TTG ACC TGT GAA** C GGT GAT GCG CCT CCA GCG CGG GGA GTT TCT TAA CCC TTT CCG ACT TAC AAG AGC CGG
$B2_{37}$	GCG AGA CTC AGG TGG TGC C TTT GGC ATT CGA CCA GGA GAT ATC GCG TTC AGC TAT GCC C
$C1_{37}$	**TTT TTT TTT TTT TTT TTT TT** C CCA TGA GAA TAA TAC CGC CGA TTT ACG TCA GTC CGG TTT CCC ACA CGG GAC GGC AGG C

**$C2_{37}$**	CGC ACA GAA CAC ATA CGC TTT GGG CAT AGC TGA ACG CGA TAT CTC CTGGTC GAA TGC C
**$D1_{37}$**	**TTG ACC TGT GAA** G CCC AGA TTA AAA CGT GAA TTC CTA GCC AGT ACA GGG TTT CCG GAC TGA CGT AAA TCG G
**$D2_{37}$**	CGG TAT TAT TCT CAT GGG TTT GGC ACC ACC TGA GTC TCG CCC GGC TCTTGT AAG TCG G
**Kim-TDF$_{37}$ for FRET assay**	
**$A1_{37}$-Cy5**	Cy5- **TTG ACC TGT GAA** C CCT GTA CTG GCT AGG AAT TCA CGT TTT AAT CTG GGC TTT GGG TTA AGA AAC TCC CCG
**$B1_{37}$-Cy5**	Cy5- **TTG ACC TGT GAA** C GGT GAT GCG CCT CCA GCG CGG GGA GTT TCT TAA CCC TTT CCG ACT TAC AAG AGC CGG
**$D1_{37}$-Cy5**	Cy5- **TTG ACC TGT GAA** G CCC AGA TTA AAA CGT GAA TTC CTA GCC AGT ACA GGG TTT CCG GAC TGA CGT AAA TCG G
**Modified DNA**	
**DNA-Pep$_{Kim-1}$**	NH_2-**TTC ACA GGT CAA**
**DNA-Rep$_{800CW}$**	NH_2- **AAA AAA AAA AAA AAA AAA AA**
**Cy3-DNA**	TTC ACA GGT CAA-Cy3 **(for FRET assay)**

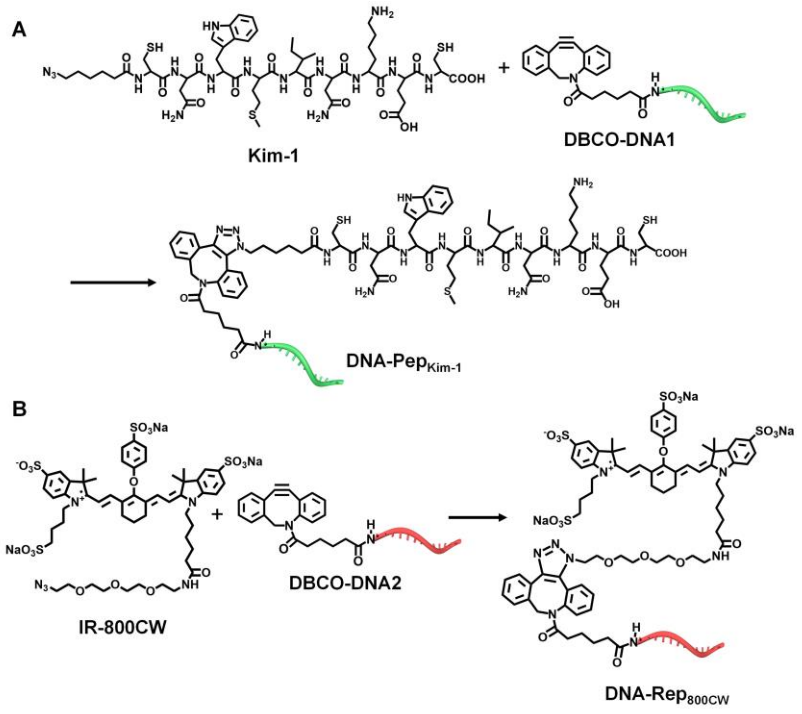

Figure S1. The illustration of synthesis of DNA-Pep$_{Kim-1}$ and DNA-Rep$_{800CW}$.

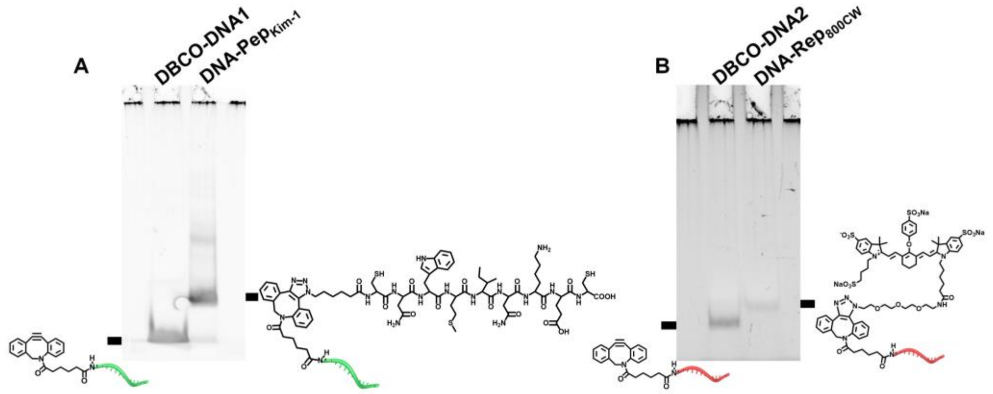

Figure S2. Characterization of DNA-Pep$_{Kim-1}$ and DNA-Rep$_{800CW}$ by 10% denaturing PAGE gel electrophoresis.

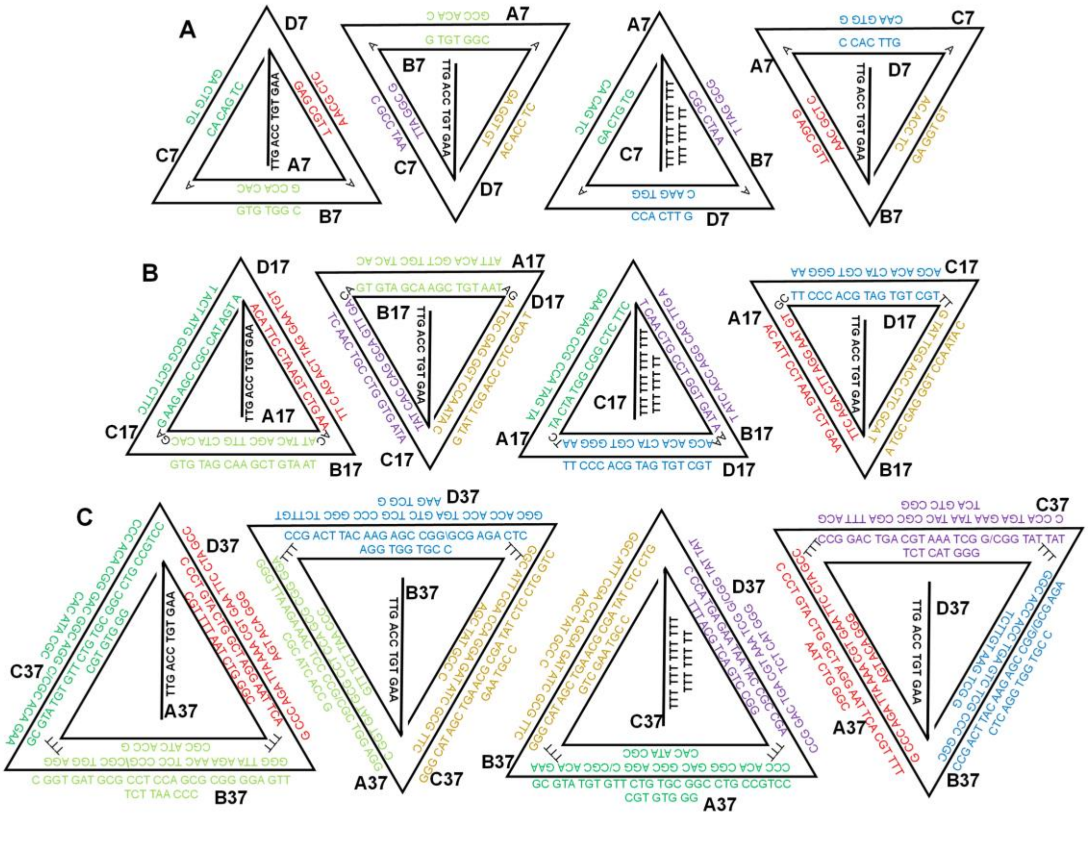

Figure S3. The sequence design of DNA strands used for the self-assembly of Kim-TDF. Each oligonucleotide was assembled with the other three to form a face of the Kim-TDF. (A) Kim-TDF$_7$; (B) Kim-TDF$_{17}$; (C) Kim-TDF$_{37}$.

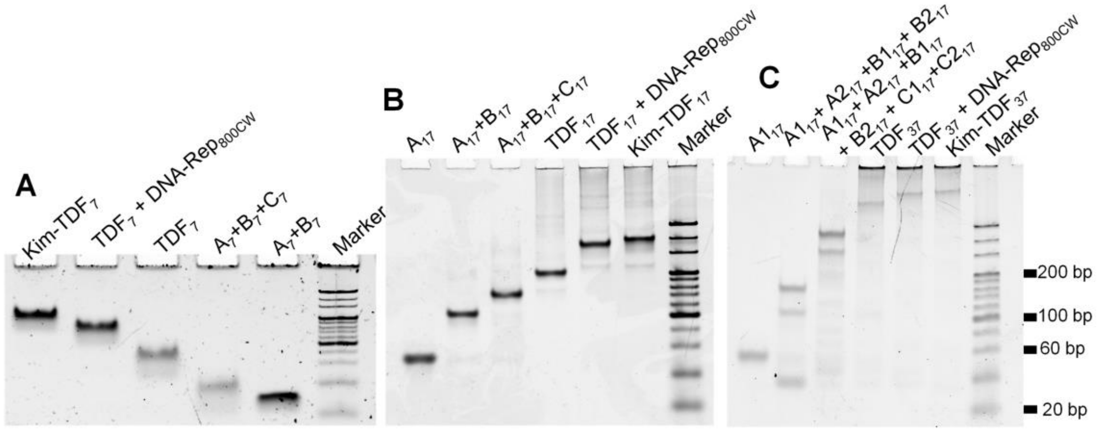

Figure S4. Characterization of Kim-TDF$_7$ (A), Kim-TDF$_{17}$ (B) and Kim-TDF$_{37}$ (C) by native PAGE gel electrophoresis.

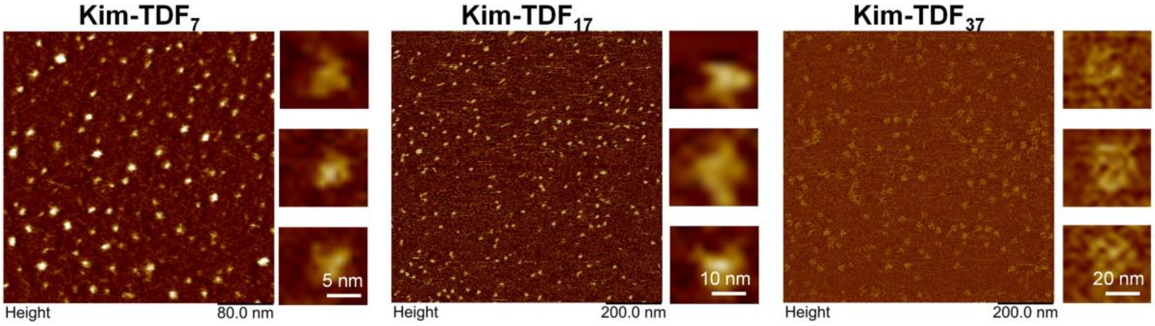

Figure S5. AFM analysis of Kim-TDF.

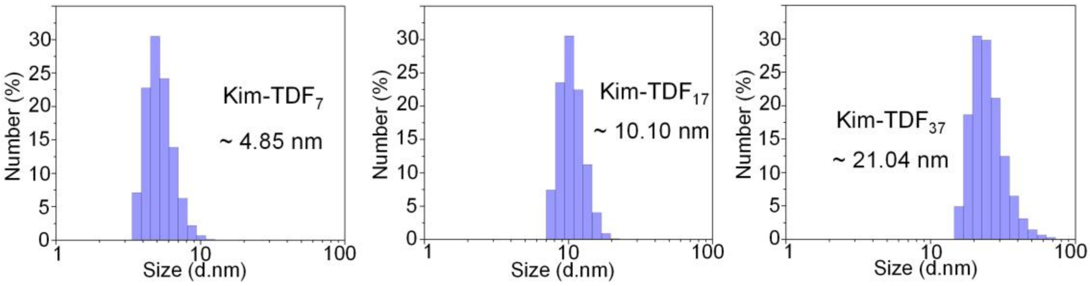

Figure S6. DLS analysis of Kim-TDFs.

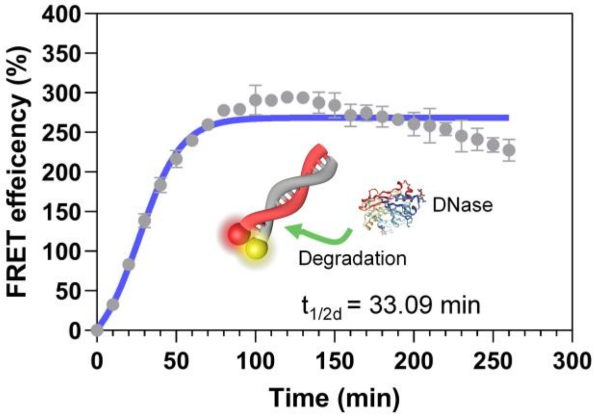

Figure S7. The degradation kinetics of double strands DNA via FRET analysis. Data represent mean ± S.D. (n = 3).

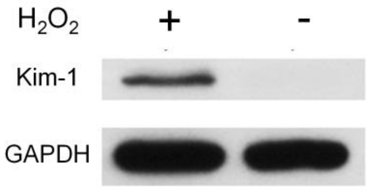

Figure S8. Western blot analysis of Kim-1 expressions in normal and H_2O_2-stimulated HK-2 cells.

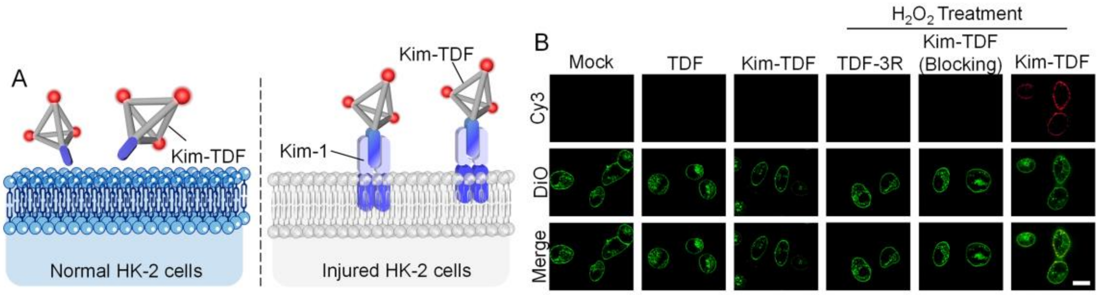

Figure S9. The Kim-1-binding ability of Kim-TDF. (A) The schematic illustration of Kim-1-binding behavior of Kim-TDF. (B) CLSM images of the HK-2 cells incubated with Cy3-labelled Kim-TDF. Cell membranes were stained with DiO. Scale bars: 30 μm.

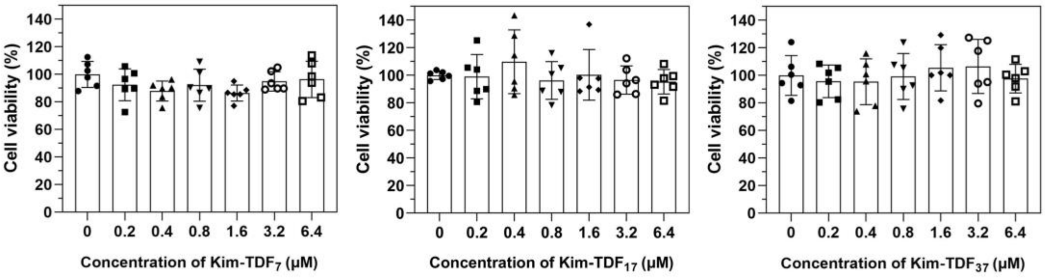

Figure S10. MTT assays of HK-2 cells treated with Kim-TDF at different concentrations. Data represent mean ± S.D. (n = 6).

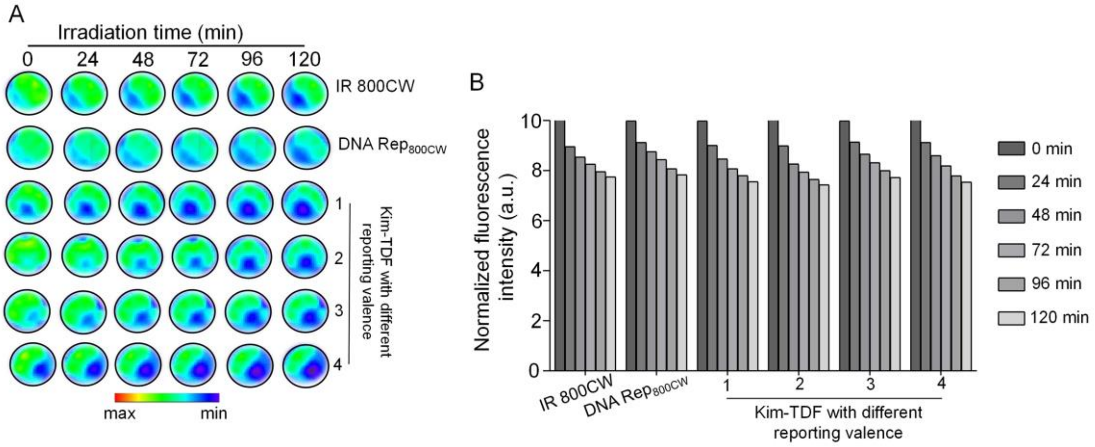

Figure S11. (A) NIR fluorescence images of different groups with equivalent fluorophore concentration (200 nM) at corresponding irradiation time points. (B) The normalized fluorescence intensity about NIR fluorescence images of different groups with equivalent fluorophore concentration (200 nM) at corresponding irradiation time points.

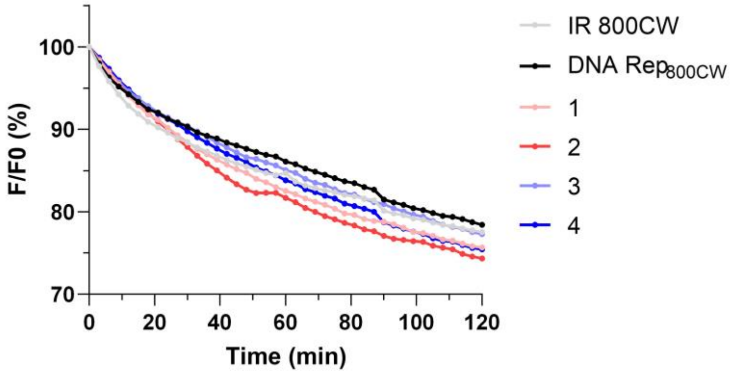

Figure S12. Quantitative fluorescence intensity changes at a specific scanning time (F0 is the initial fluorescence intensity and F is the fluorescence intensity). 1, 2, 3, 4 means the reporting valence of nanodevice.

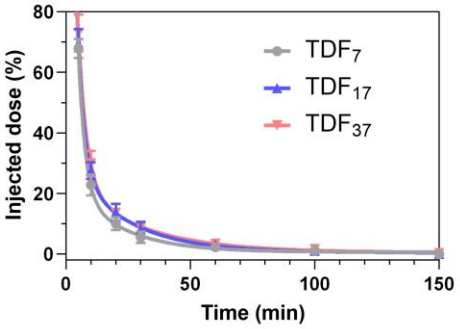

	$t_{1/2\alpha}$ (min)	$t_{1/2\beta}$ (min)
TDF7	1.84	13.69
TDF17	1.90	15.86
TDF37	2.37	19.09

Figure S13. Blood concentration (% ID) decay of TDF in healthy mice. Data represent mean ± S.D. (n = 4).

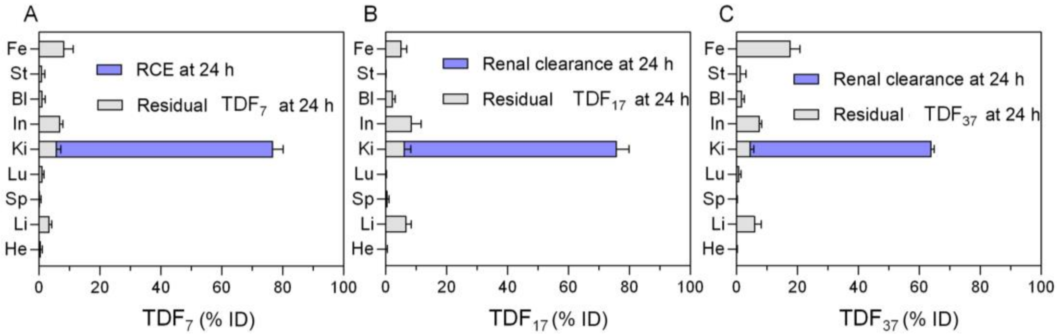

Figure S14. Analysis of the amount of TDFs excreted from kidneys into urine (blue bar) and residual TDFs in major organs of mice (gray bar) after 24 h injection of TDFs. Major organs were homogenized in PBS and centrifuged to remove insoluble components. The supernatant containing extracted TDFs were analyzed. Heart (He),

liver (Li), spleen (Sp), lung (Lu), kidneys (Ki), intestine (In), bladder (Bl), stomach (St), feces (Fe). Data represent mean ± S.D. (n = 4).

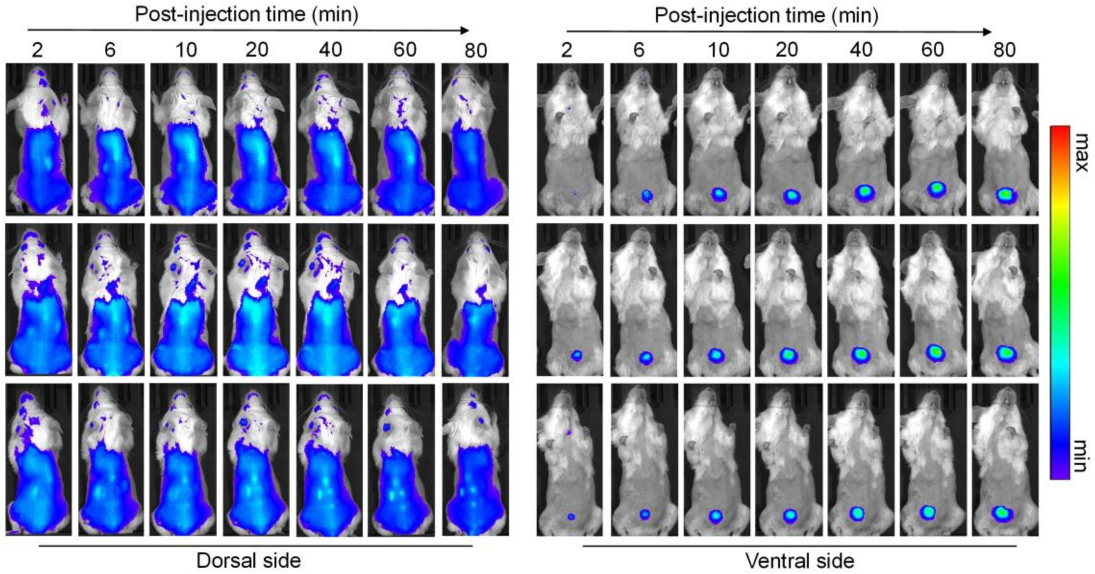

Figure S15. NIR images of healthy mice at different post-treatment time points of Kim-TDF_7.

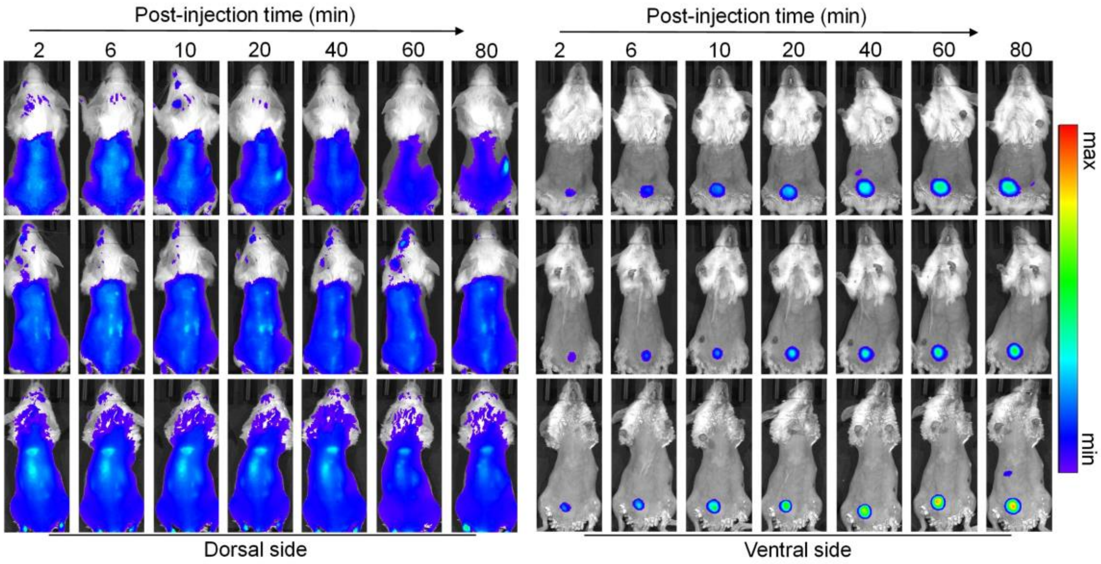

Figure S16. NIR images of healthy mice at different post-treatment time points of Kim-TDF_{17}.

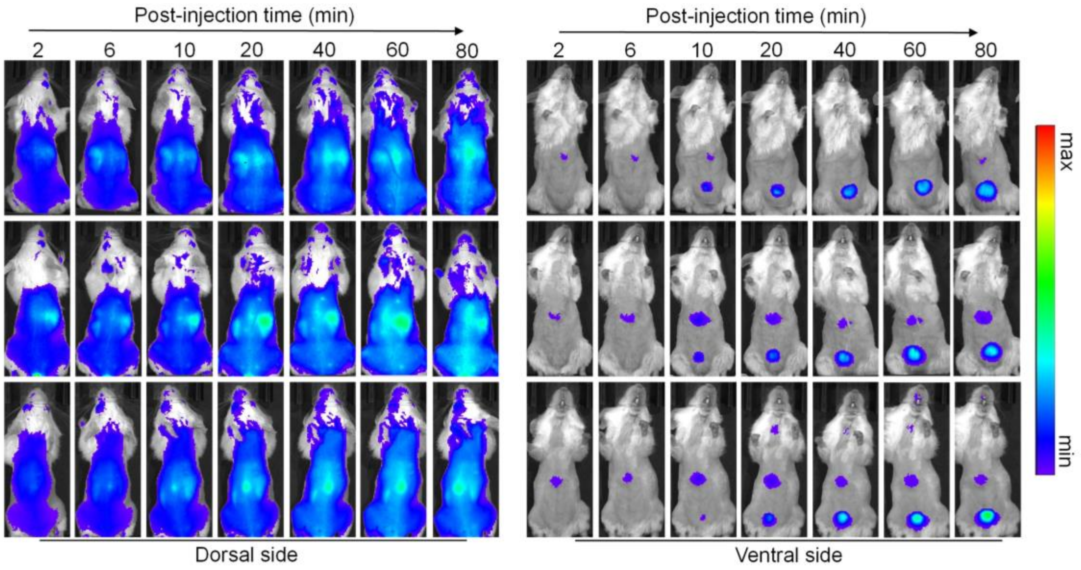

Figure S17. NIR images of healthy mice at different post-treatment time points of Kim-TDF$_{37}$.

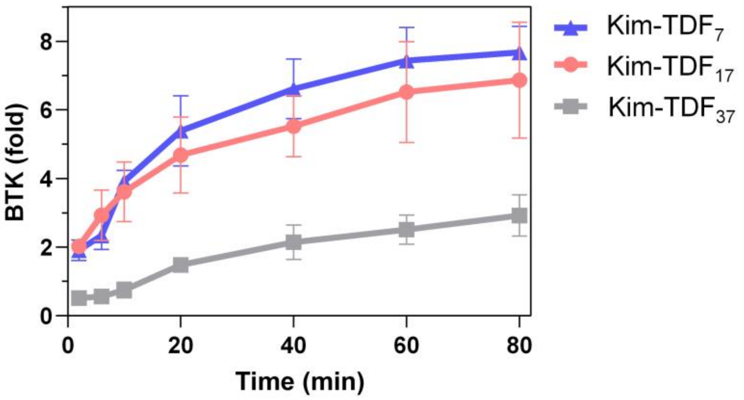

Figure S18. The ratios of bladder-to-kidney intensity of Kim-TDFs as a function of time post-injection of Kim-TDFs in healthy mice. Data represent mean ± S.D. (n = 3).

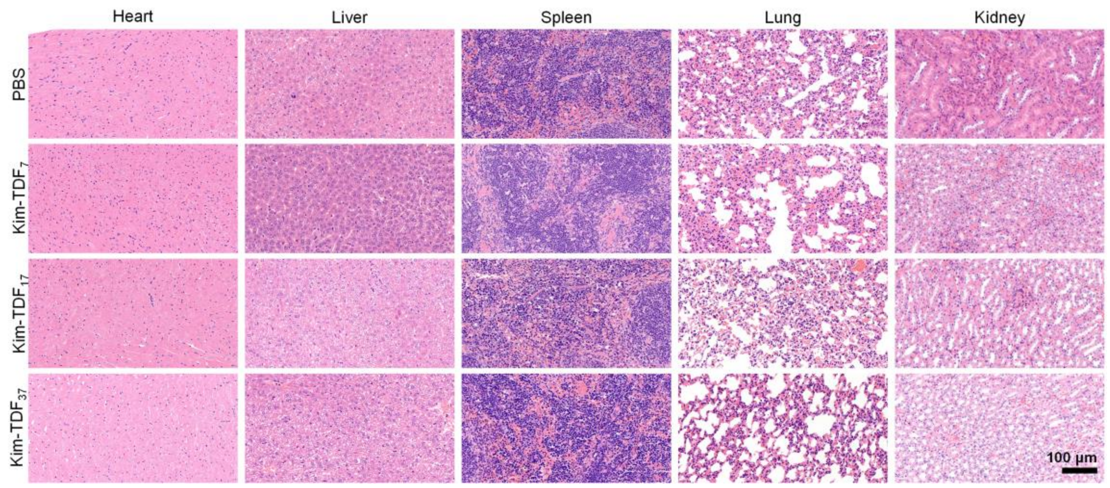

Figure S19. Microscopic images of H&E-stained sections of the major organs after treatment with Kim-TDF.

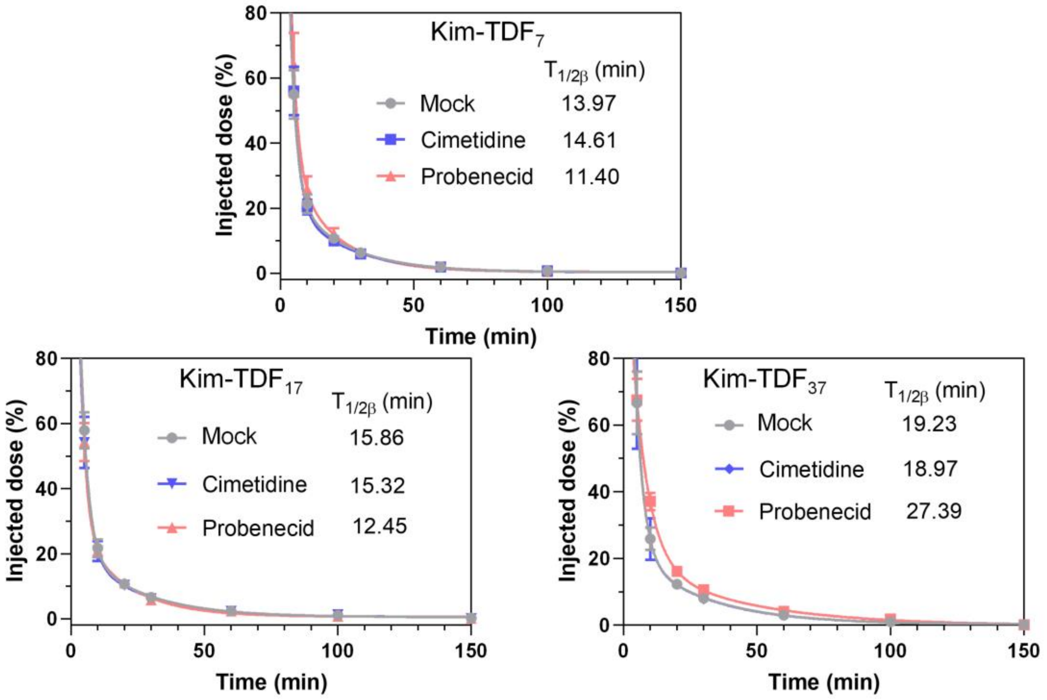

Figure S20. Blood concentration (% ID) decay of Kim-TDFs in living mice pretreated with probenecid or cimetidine. Data represent mean ± S.D. (n = 4).

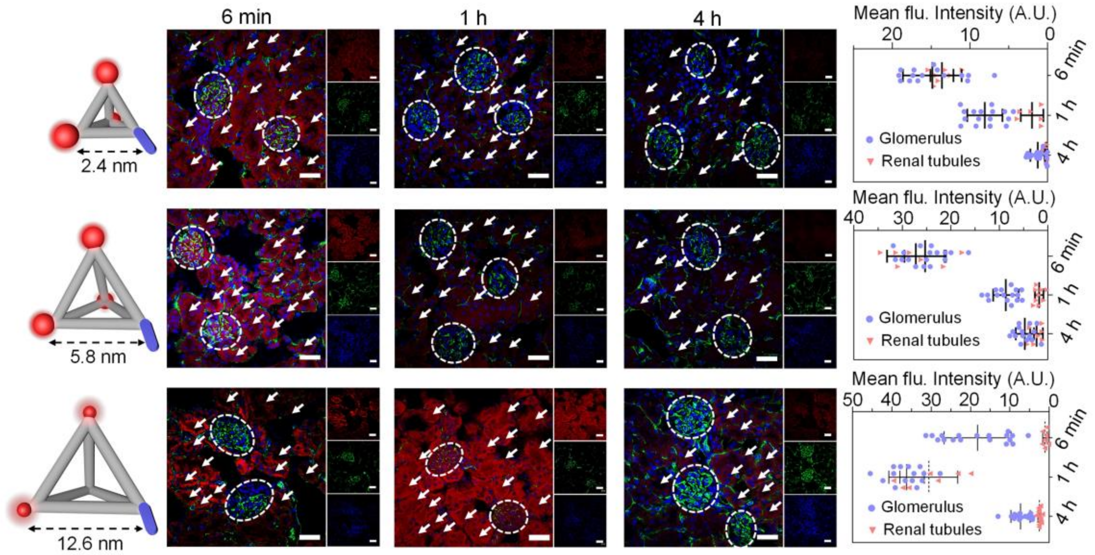

Figure S21. Fluorescence images of glomerulus and tubules at tissue level at 6 min, 1 h and 4 h post-injection of Cy3-labelled Kim-TDFs (red signal). Nuclei were stained with 2-(4-amidinophenyl)-1H-indole-6-carboxamidine (DAPI; blue). Blood vessel stained with anti-CD31 antibody (green). Arrows denote renal tubules, and the circles denote glomerulus.

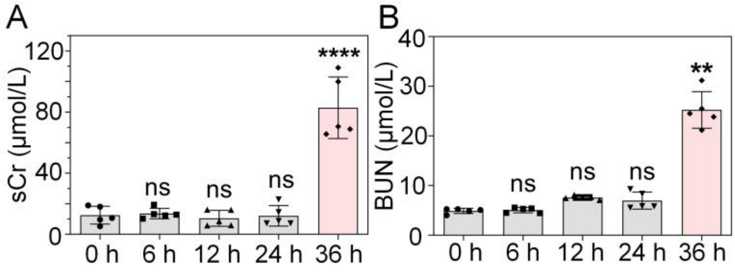

Figure S22. Change in sCr, BUN in living mice at different time points post-induction with 50 % glycerol. Data represent mean ± S.D. (n = 5). Statistical analysis in 0 h versus other groups; **p<0.01, ****p<0.0001; ns, no significance.

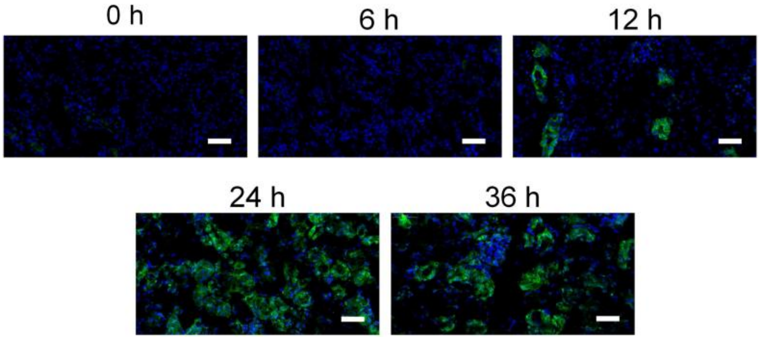

Figure S23. Anti-Kim-1 analysis of kidney at different time points post-treatment with 50 % glycerol. Scale bars: 50 μm.

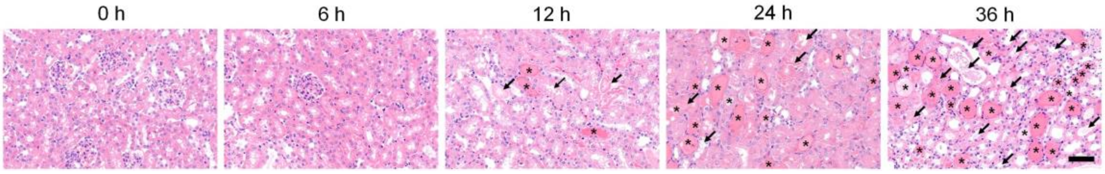

Figure S24. Representative photomicrographs of H&E staining in paraffin-embedded kidney sections from mice 0, 6, 12, 24 or 36 h after 50 % glycerol induction. Arrows denote damaged tubules, and asterisks denote the formation of casts-structures formed via precipitation of denatured proteins in the tubules. Scale bar: 50 μm.

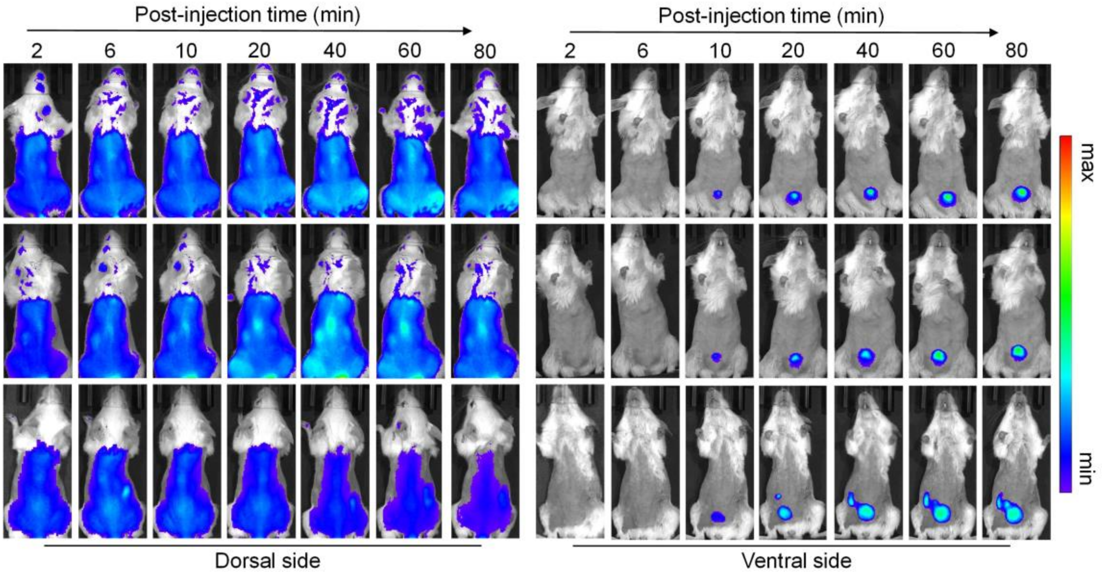

Figure S25. NIR fluorescence images of living mice after injection of Kim-TDF$_7$ at 12 h post-treatment with 50 % glycerol.

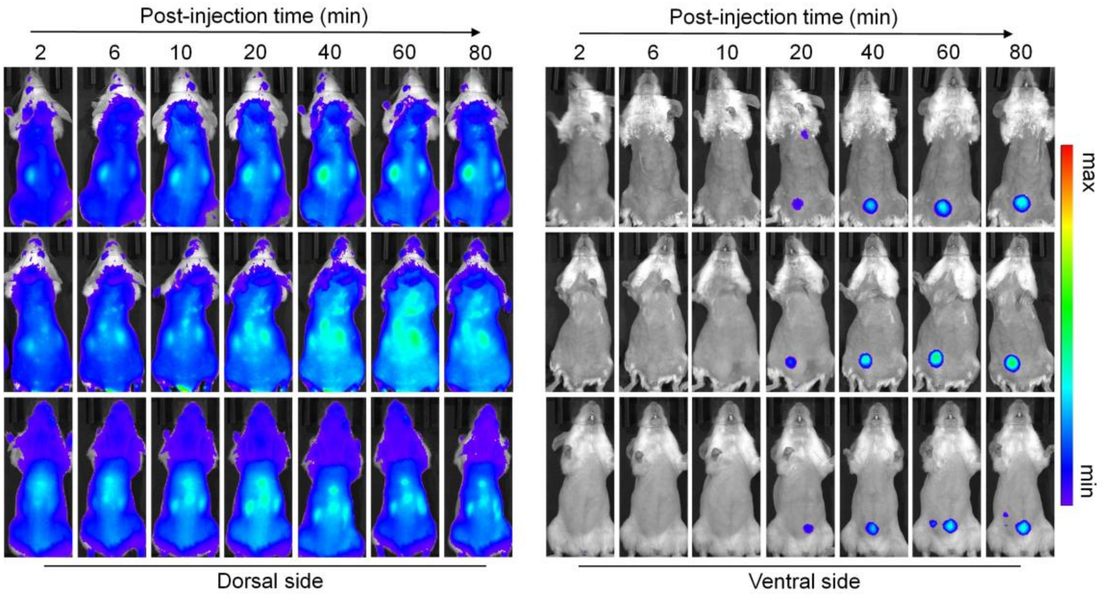

Figure S26. NIR fluorescence images of living mice after injection of Kim-TDF$_{17}$ at 12 h post-treatment with 50 % glycerol.

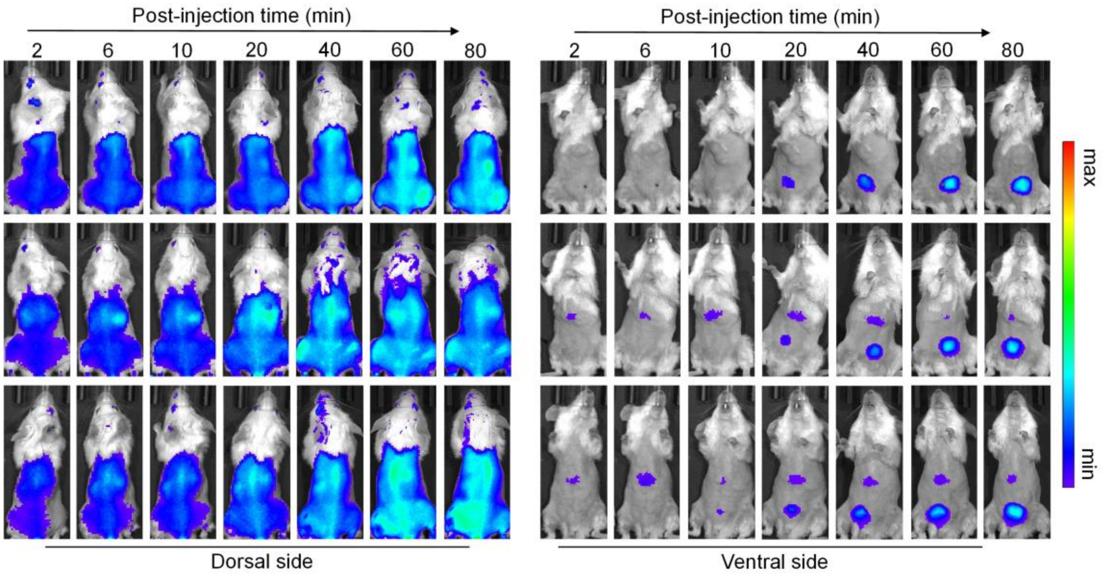

Figure S27. NIR fluorescence images of living mice after injection of Kim-TDF$_{37}$ at 12 h post-treatment with 50 % glycerol.

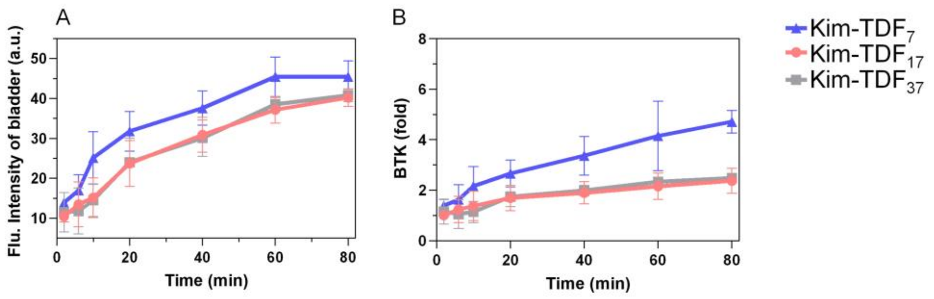

Figure S28. (A) NIR fluorescence intensities of bladder at different post-injection time points of Kim-TDFs in living mice at 12 h post-induction with 50 % glycerol. (B) The ratios of bladder-to-kidney intensity at different post-injection time points of Kim-TDFs in living mice at 12 h post-induction with 50 % glycerol. Data represent mean ± S.D. (n = 3).

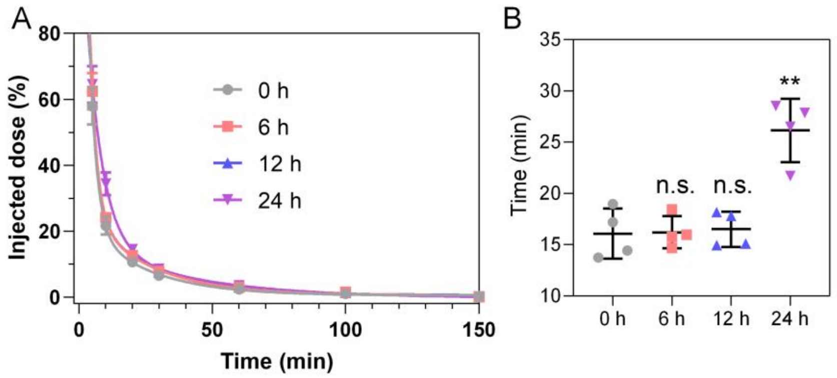

Figure S29. (A) Blood concentration (% ID) decay of Kim-TDF$_{17}$ in living mice at 0, 6, 12, 24 h post-induction with 50 % glycerol. (B) The $t_{1/2\beta}$ of Kim-TDF$_{17}$ in mice at 0, 6, 12, 24 h post-induction with 50 % glycerol. Data represent mean± S.D. (n = 4). Statistical analysis in (B) 0 h versus other groups; mean ± S.D.; **p<0.01; ns, no significance.

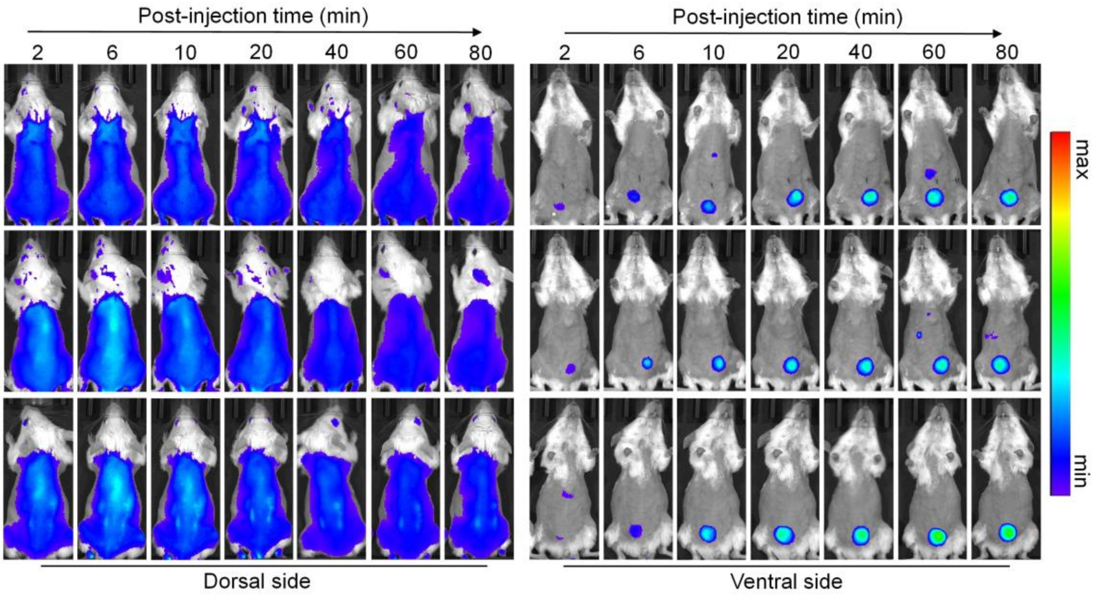

Figure S30. NIR fluorescence images of living mice after injection of Kim-TDF_{17} at 6 h post-treatment with 50 % glycerol.

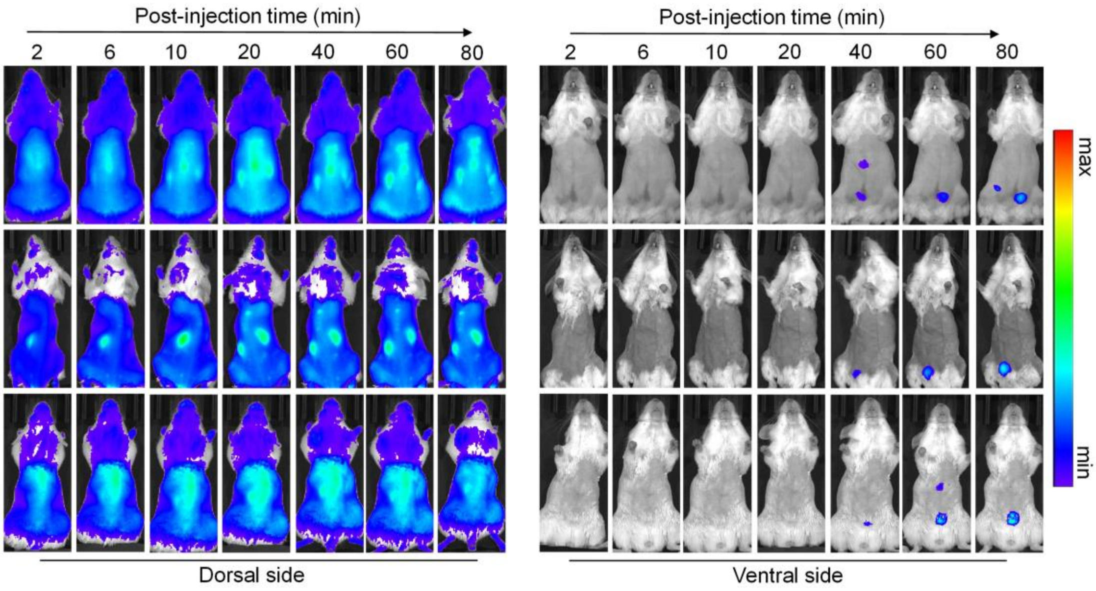

Figure S31. NIR fluorescence images of living mice after injection of Kim-TDF_{17} at 24 h post-treatment with 50 % glycerol.

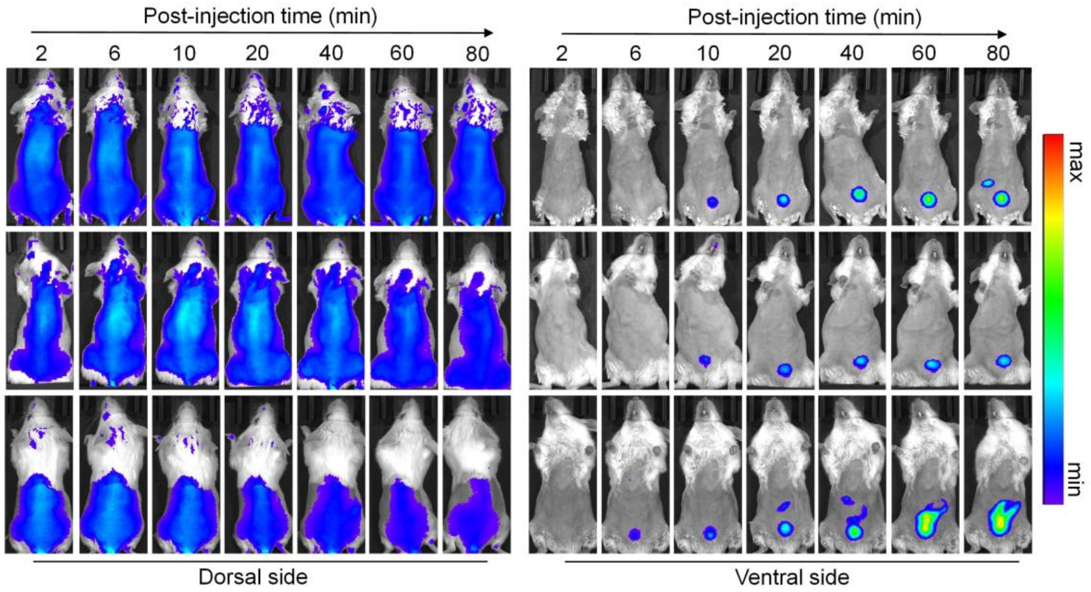

Figure S32. NIR fluorescence images of living mice after injection of TDF_{17}-3R at 12 h post-treatment with 50 % glycerol.

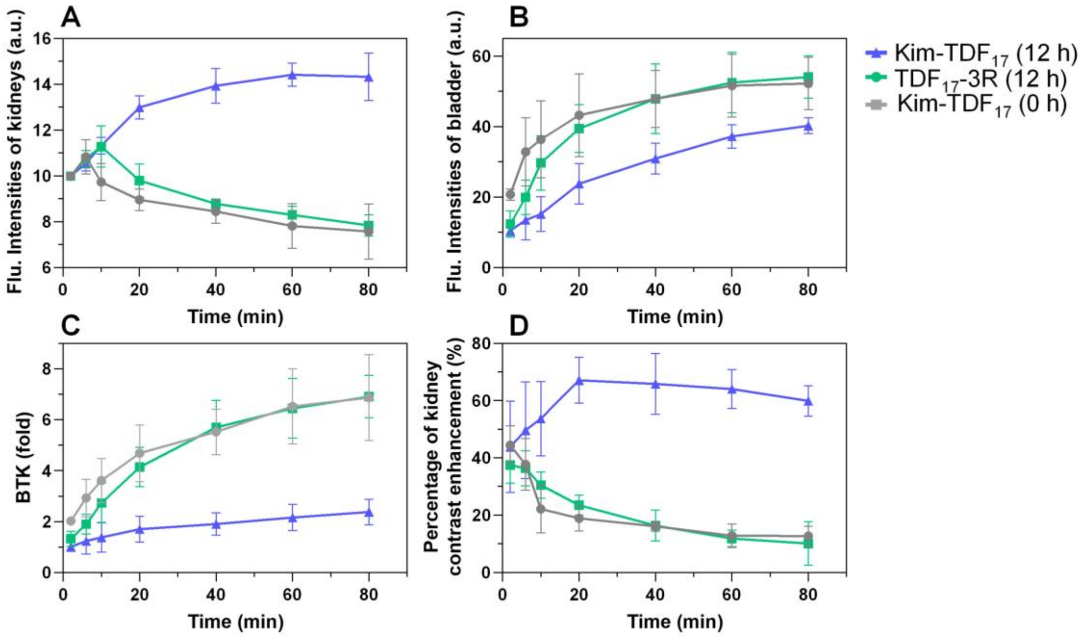

Figure S33. (A) NIR fluorescence intensities of kidneys and B) bladder at different post-injection time points of TDF_{17}-3R in living mice at 12 h post-induction with 50 % glycerol. (C) The ratios of bladder-to-kidney intensity and D) percentage of kidney contrast enhancement at different post-injection time points of TDF_{17}-3R in living mice at 12 h post-induction with 50 % glycerol. Data represent mean ± S.D. (n = 3).

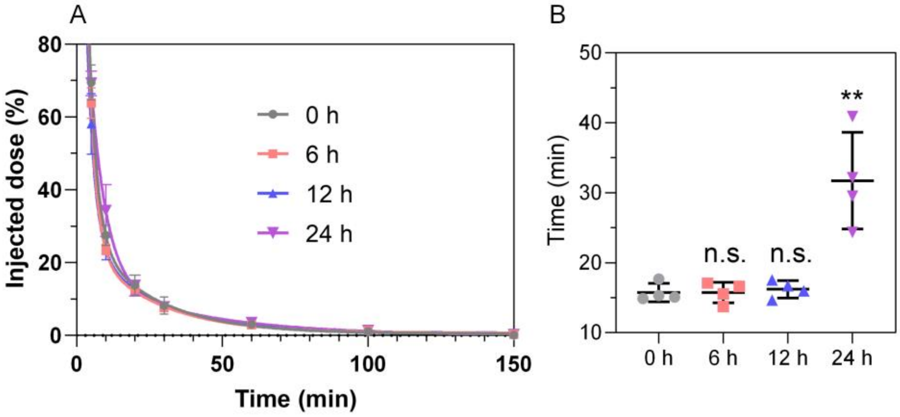

Figure S34. (A) Blood concentration (% ID) decay of TDF_{17}-3R in living mice at 0, 6, 12, 24 post-induction with 50 % glycerol. (B) The $t_{1/2\beta}$ of TDF_{17}-3R in mice at 0, 6, 12, 24 post-induction with 50 % glycerol. Data represent mean± S.D. (n = 4). Statistical analysis in (B) 0 h versus other groups; mean ± S.D.; **p<0.01; ns, no significance.
